# The Use of Stem Cells as a Potential Treatment Method for Selected Neurodegenerative Diseases: Review

**DOI:** 10.1007/s10571-023-01344-6

**Published:** 2023-04-07

**Authors:** Elżbieta Cecerska-Heryć, Maja Pękała, Natalia Serwin, Marta Gliźniewicz, Bartłomiej Grygorcewicz, Anna Michalczyk, Rafał Heryć, Marta Budkowska, Barbara Dołęgowska

**Affiliations:** 1 Department of Laboratory Medicine, Pomeranian Medical University of Szczecin, PowstancowWielkopolskich 72, 70-111 Szczecin, Poland; 2 Department of Psychiatry, Pomeranian Medical University of Szczecin, Broniewskiego 26, 71-460 Szczecin, Poland; 3 Department of Nephrology, Transplantology and Internal Medicine, Pomeranian Medical University of Szczecin, PowstancowWielkopolskich 72, 70-111 Szczecin, Poland; 4 Department of Medical Analytics, Pomeranian Medical University of Szczecin, PowstancowWielkopolskich 72, 70-111 Szczecin, Poland

**Keywords:** Amyotropic lateral sclerosis, Multiple sclerosis, Parkinson's disease, Huntington's disease, Alzheimer's disease

## Abstract

Stem cells have been the subject of research for years due to their enormous therapeutic potential. Most neurological diseases such as multiple sclerosis (MS), amyotrophic lateral sclerosis (ALS), Alzheimer's disease (AD), Parkinson's disease (PD), and Huntington's disease (HD) are incurable or very difficult to treat. Therefore new therapies are sought in which autologous stem cells are used. They are often the patient's only hope for recovery or slowing down the progress of the disease symptoms. The most important conclusions arise after analyzing the literature on the use of stem cells in neurodegenerative diseases. The effectiveness of MSC cell therapy has been confirmed in ALS and HD therapy. MSC cells slow down ALS progression and show early promising signs of efficacy. In HD, they reduced huntingtin (Htt) aggregation and stimulation of endogenous neurogenesis. MS therapy with hematopoietic stem cells (HSCs) inducted significant recalibration of pro-inflammatory and immunoregulatory components of the immune system. iPSC cells allow for accurate PD modeling. They are patient—specific and therefore minimize the risk of immune rejection and, in long-term observation, did not form any tumors in the brain. Extracellular vesicles derived from bone marrow mesenchymal stromal cells (BM-MSC-EVs) and Human adipose-derived stromal/stem cells (hASCs) cells are widely used to treat AD. Due to the reduction of Aβ42 deposits and increasing the survival of neurons, they improve memory and learning abilities. Despite many animal models and clinical trial studies, cell therapy still needs to be refined to increase its effectiveness in the human body.

**Graphical Abstract:**

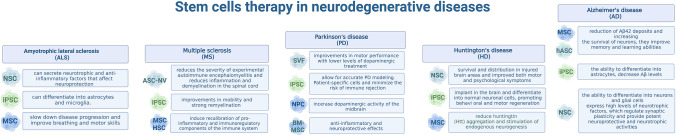

## Stem Cells

All developed tissues and organs arise from stem cells (SC). This type of cell can self-renewal and divide over a long period. However, they are only specialized once they receive the appropriate signal (Kacperska et al. 2013; Dulak 2020). Adult stem cells (ASCs) can transform into only one cell type, such as skeletal muscle cells or epidermis, or make one cell type (from a single germ layer). Moreover, they can differentiate into many kinds of cells derived from all three germ layers (ectoderm, endoderm, and mesoderm), the areas of the embryo that form during development. Based on the differentiation potential, stem cells can be divided into totipotent, pluripotent, multipotent, and unipotent cells (Dulak 2020).

**Fig. 1:**
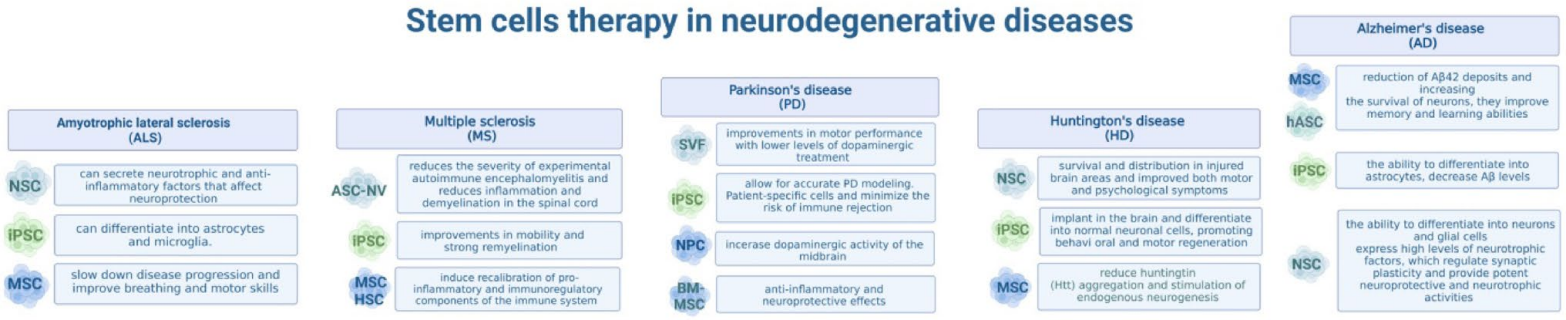
The use of stem cells in the treatment of neurodegenerative diseases. The use of stem cells in the treatment of diseases of the five neurodegenerative diseases (ALS, MS, PD, HD, AD) shows promising results even a few months after the therapy. Through neuroprotective effects, stem cells allow delaying the progression of the disease and immunomodulatory effect. However, the use of the same type of cells has a different therapeutic effect depending on the type of disease, e.g., the use of mesenchymal stem cells (MSCs) in ALS therapy improves breathing and motor skills, while in AD therapy they improve memory and learning abilities. Current research using these cells and the opportunities and risks of future therapies are described in detail in this review

Pluripotent stem cells (PSCs) multiply indefinitely and differentiate into cells of all three germ layers. These two properties make PSCs an attractive source of cell therapies for various diseases and injuries. Particularly relevant for clinical use are embryonic stem cells (ESCs) and induced pluripotent stem cells (iPSCs) (Yamanaka 2020). The first human ESC lines were established from donated human embryos, while because of a limited supply of donor embryos, human ESCs derivation remains ethically and politically controversial (Kolagar et al. 2020). While these cells raise ethical and immunological concerns, the translation of human ESC research into clinical practice has begun. The generation of human induced pluripotent stem cell (iPSC) -like embryonic stem cells (iPSCs) from somatic cells by virus-mediated overexpression of different sets of reprogramming factors (OCT4, SOX2, KLF4, and c-MYC or OCT4, SOX2, NANOG, and LIN28) in 2007 opened further opportunities in this field. Bypassing the major controversies surrounding human ESCs, induced pluripotent stem cells offer the same benefits and new perspectives for personalized medicine (Drews et al. 2012) (see Fig. 1).

Induced pluripotent stem cells (iPSCs) have the same properties as ESCs. Therefore, hiPSC-based therapies effectively replace human ESCs without destroying embryos. Compared to invasive methods for obtaining human ESCs, human iPSCs enabled the reprogramming patient-specific cells with specific factors and minimally invasive procedures.They are also obtained exclusively in the laboratory. The method was developed first on a mouse model, then a human model. This method involves introducing active genes into somatic cells (they were fibroblasts), thanks to which these cells are reprogrammed to their original state. As a result, within a dozen or so days, pluripotent cells appear, but, like germ cells, iPSCs do not form trophoblasts. The properties of these cells have been confirmed in tests analogous to ESC—mouse and human iPSC can efficiently differentiate in vitro into many types of cells. Human pluripotent stem cells are a good source for cell research, cell replacement therapies, and disease modeling. Hundreds of human ESC and iPSC lines have been generated to treat various neurodegenerative diseases (Kolagar et al. 2020).

Stem cells with high potential for use in neurodegenerative diseases are certainly NSCs. Apart from MSCs, they are the most widely used in clinical trials. NSC refers to an uninvolved cell with the potential to differentiate into neurons and CNS glia. NSC is defined by two fundamental characteristics: self-renewal and multipotency. During development, neural stem cells (NSCs) form the central nervous system. Initially, NSCs, also called neuroepithelial cells, differentiate into radial glial cells and proliferate into pools of neural progenitor cells (NPCs) (Finkel et al. 2021; Kawaguchi et al. 2020). These neural stem and progenitor cells (NSPCs) represent both populations and are established as the only self-renewing cell type in the adult CNS. NSPCs migrate and differentiate into highly defined networks of neurons through neurogenesis, and oligodendrocytes and astrocytes are generated by gliogenesis (Bond et al. 2020). Thus, NSPCs are the main line of research in regenerative medicine. Extrinsic and intrinsic factors such as neurotrophic/growth factors, transcription factors, and canonical pathways guide neurogenesis and gliogenesis during development and adulthood. Neural stem cell (NSC) transplantation has provided the basis for developing potentially powerful new cell-based therapeutic strategies for a broad spectrum of clinical conditions, including stroke and mental illnesses such as fetal alcohol disorders and cancer (Tuazon et al. 2019).

Adult stem cells, also known as somatic stem cells, have a high proliferative potential and can differentiate into different cell types depending on the tissue from which they originate. Adult stem cells produce new tissue in response to injury, disease, or regular maintenance (Cable et al. 2020). It is also a rare population of undifferentiated cells located in a differentiated organ, in a specialized niche that maintains the microenvironments that regulate the growth and development of adult stem cells. Adult stem cells are self-renewing, clonogenic, and multipotent; their primary role is maintaining tissue homeostasis. They can be activated to proliferate and differentiate into the required cell type after cell loss or tissue damage. Adult stem cells have been identified in many tissues, including blood, intestines, skin, muscles, brain, and heart. Extensive preclinical and clinical studies have demonstrated these adult stem cells' structural and functional regenerative potential, such as bone marrow-derived mononuclear cells, hematopoietic stem cells, mesenchymal stromal/stem cells, resident adult stem cells, induced pluripotent stem cells, and umbilical cord stem cells (Gurusamy et al. 2018). Therefore, adult stem cell-based therapies have gained a lot of interest for treating various degenerative diseases and rejuvenating aging tissue. However, their proliferative nature also makes them dangerous. Dysregulation of the mechanisms that keep stem cells quiescent without increasing can lead to cancer (Cable et al. 2020).

A population of multipotent stromal cells exists within the bone marrow and other adult tissues, which can differentiate into different skeletal tissues such as bone, cartilage, and fat (mesenchymal stem cells—MSCs).MSC can be obtained from various sources, i.e., bone marrow (BM-MSC), adipose tissue (AT-MSC), embryonic tissue (E-MSC), cord blood (CB-MSC), reprogramming of mature cells (iMSC) and perinatal tissue-Wharton’s jelly (WJ-MSC) (Frausin et al. 2015) and amniotic membrane (Hass et al. 2011). They offer significant therapeutic potential, particularly in orthopedic applications. They may also have broader roles in regenerative medicine and cancer treatment as anti-inflammatories, immunosuppressives, and vehicles for gene/protein therapy (Cook and Genever 2013). What's more, they do not cause teratomas. They are easy to isolate and have the ability to differentiate multipotent. What is essential is that the conversion of cells to any other cell type under laboratory conditions must be handled with care. The efficiency of such a process is crucial (very often low in the case of MSC cells). There is no evidence or justification that MSC cells, when administered undifferentiated into the blood or tissue, will transform into the type of cells necessary for repairing the damaged organ. They can be differentiated into undesirable mesenchymal lines, e.g., through transformations and cytogenetic aberrations, which may harm their therapeutic application (Dulak 2020; Ayala-Cuellar, et al. 2019; Karussis et al. 2010).

The narrowest differentiation possibilities and the unique property of multiple division characterize unipotent stem cells. The latter feature makes them a promising candidate for therapeutic use in regenerative medicine (Zakrzewski et al. 2019). These cells can only form one type of mature cells (e.g., epithelial cells), retaining the ability to divide compared to adult cells. In the central nervous system (CNS), they are found, for example, in the olfactory epithelium and ependyma, when nerve tissue damage is acute or chronic. However, it is interesting that in elderly patients, e.g., after an ischemic stroke, there is often a significant improvement in the clinical picture, even without primary therapeutic intervention. Therefore, stem cells are efficient and productive not only in young people. In animal models, new therapies have started to be developed that use human SCs. Promising results have been obtained in treating neurodegenerative diseases such as amyotrophic lateral sclerosis, Parkinson's disease, and Huntington's disease (BogusławMachaliński 2008).

**Fig. 2:**
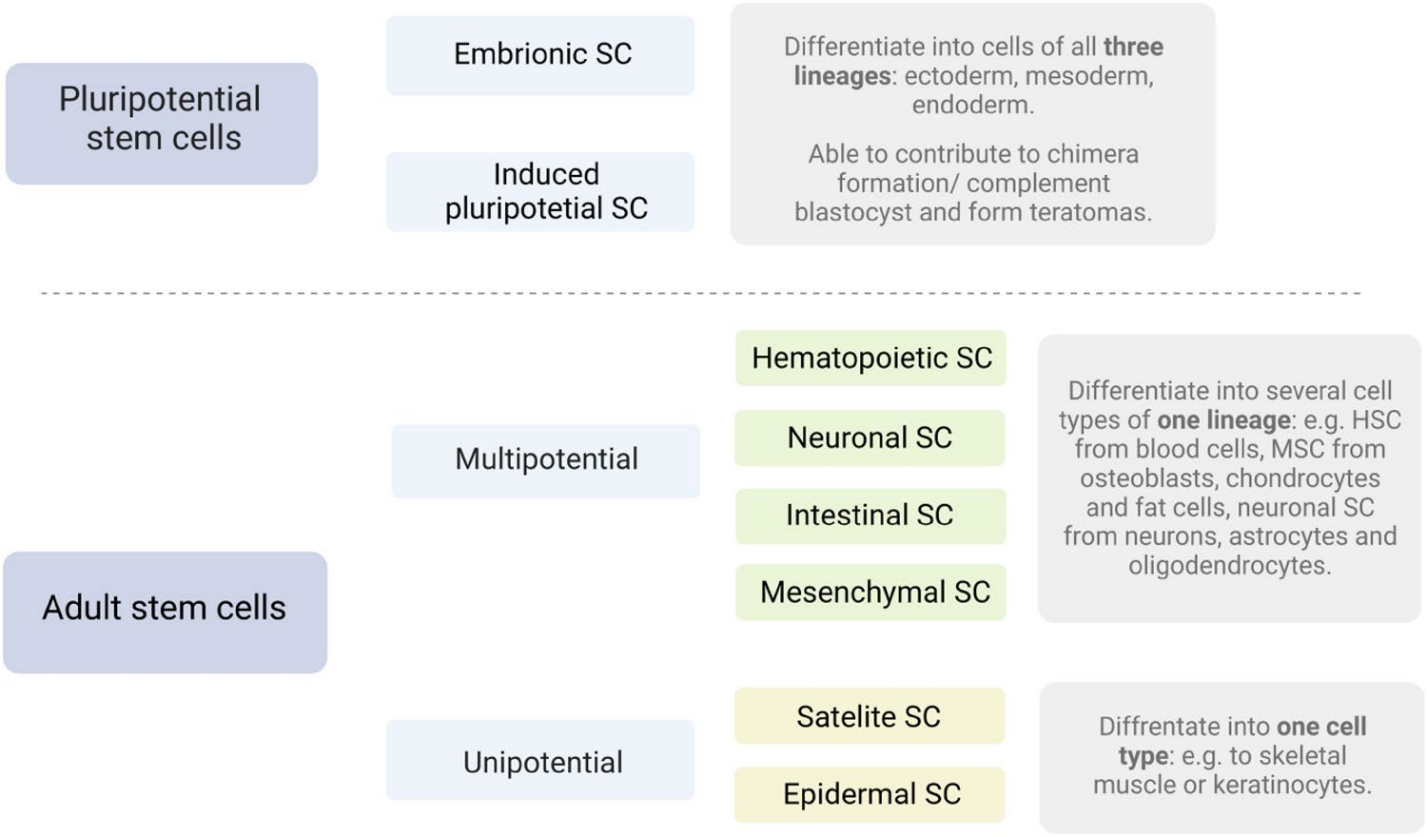
Division of stem cells based on differentiation potential. Stem cells can be divided based on their ability to differentiate. The two main groups are pluripotent cells and cells found in the adult organism. Under normal conditions, pluripotent stem cells do not exist in adult organisms—they are obtained by separating the inner cell mass of blastocysts (embryonic stem cells). They are able to differentia te into cells of all three cell lineages. Adult stem cells are either pluripotent or unipotent. Multipotent stem cells are able to differentiate into several cell types of one lineage, while unipotent stem cells differentiate into one specific cell type. Both pluripotent and unipotent stem cell populations can be present in the same organ, such as the skin

This review aims to show that thanks to stem cell therapy, it is possible not only to delay the progression of incurable neurodegenerative diseases such as Parkinson's disease, Alzheimer'sdisease (AD), and Huntington's disease but, above all, to remove the source of the problem. The discovery of NSCs has refuted the previous view that the adult CNS is incapable of neurogenesis (Ma et al. 2009; Dantuma et al. 2010). Neural stem cells can improve cognition in preclinical AD models in rodents (Byrne 2014). In turn, iPSCs were obtained from skin biopsies to develop a neural stem cell-based approach to AD treatment. In turn, dopaminergic neurons can be efficiently generated from hESCs and used in Parkinson's disease. PD is also an ideal disease for iPSC-based cell therapy (Peng and Zeng 2011). However, this therapy is still in the experimental phase (https://www.ncbi.nlm.nih.gov/pmc/articles/PMC4539501/). While the results are controversial so far, they also demonstrate that pure stem cell therapies are an essential and increasingly affordable treatment option (Zakrzewski et al. 2019) (see Fig. 2).

## Neurodegenerative Diseases

Neurodegeneration, or the loss of nerve cells, is the cause of many neurological diseases. They can be both congenital and acquired, such as Parkinson's disease, Alzheimer's disease, Huntington's disease, multiple sclerosis, amyotrophic lateral sclerosis, or spinal muscular atrophy. In all these diseases, different nerve cell loss has been observed.

**Fig. 3:**
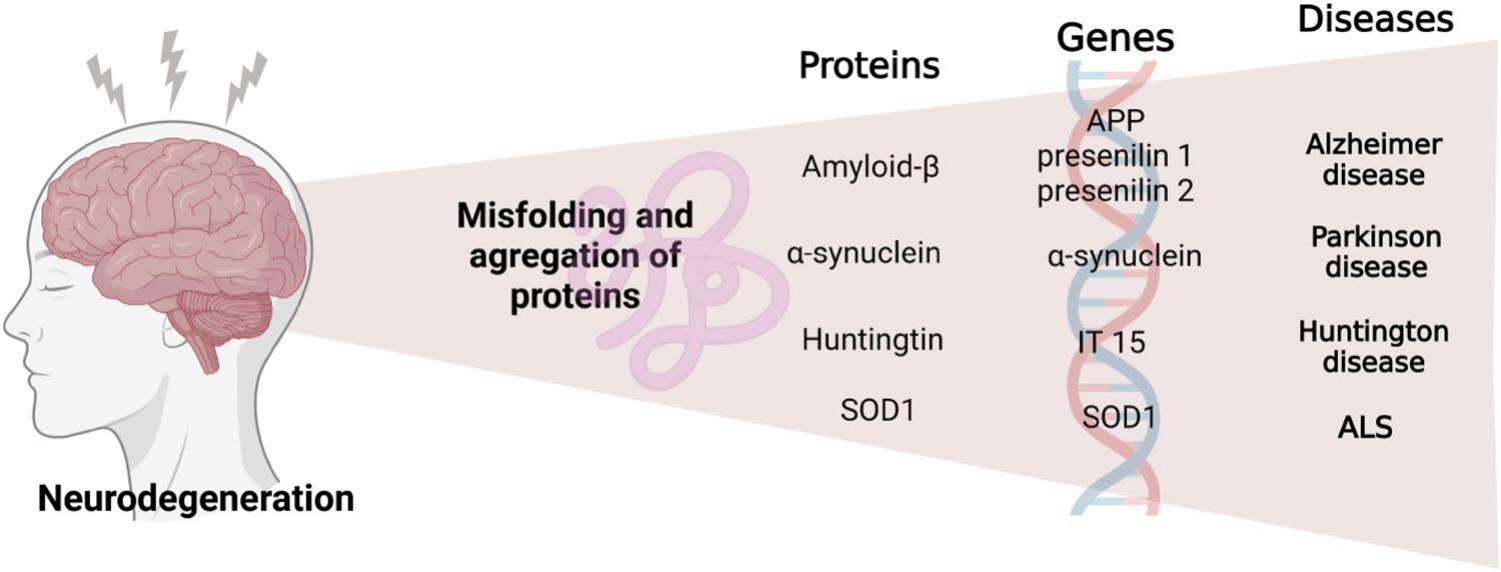
The neurodegenerative process. Neurodegeneration is a process in which nerve cells become damaged, leading to dysfunction of the entire nervous system. Damaged neurons work less well and are unable to communicate with other neurons with normal efficiency, and eventually die. It may be caused by gene dysfunctions which disrupt the production of normal protein, resulting in a protein with a disordered structure that does not perform its function e.g., IT 15 gene encodes the huntingtin protein which aggregates in the nuclei of neurons, thereby inhibiting the action of various proteins, including key transcription factors

In most neurodegenerative diseases, this process is slow and takes months or even years. Brain aging is the leading cause of cell degeneration. Still, this process can be accelerated by many factors, such as mitochondrial dysfunction, oxidative stress, activation of apoptosis (programmed cell death), or neurological inflammation. The formation of protein aggregates, localized neuronal death, and continually progressive symptoms are typical of neurodegeneration. However, the knowledge of the pathogenesis and etiology underlying these disorders is still being researched in various fields of genetics. However, early diagnosis and therapy are the most effective way to fight the disease. There is a growing interest in regenerative medicine, and research into personalized medicine is ongoing to stop or slow the progress of these diseases (Surma and KamilMaciąg 2020; Stoddard-Bennett and Reijo Pera 2019) (see Fig. 3).

### Amyotrophic Lateral Sclerosis

Amyotrophic lateral sclerosis (ALS) is a neurodegenerative disease that selectively affects motor neurons of the brain and spinal cord. Over time, central (cerebral) and peripheral (spinal) motor neurons disappear, gradually denervating to muscle loss. The axons of these neurons are considered the longest in our body and can start in the brain's cortex and extend to the sacral segments of the spinal cord. In the United States, 6000 new patients are diagnosed with ALS yearly. The prevalence of ALS is 5.0 per 100,000 of the US population, with approximately 16,000 to 20,000 individuals identified with definite ALS, with a prognosis for survival of 2–5 years. The highest prevalence of ALS is in whites, males, and people 60 years or older. In European populations, prevalence is ~ 5/100,000 people and a mean age of onset of ~ 64 years (lifetime risk ~ 1:400). Globally, the mean age of ALS onset is 62 years (Hulisz 2018; Chio et al. 2013; Kiernan et al. 2011; Mehta et al. 2018). There are two primary classifications of ALS: sporadic (idiopathic) and familial. Familial ALS occurs in about 5% to 10% of patients with ALS, usually due to a dominant traitSporadic ALS encompasses all other patients with ALS. The affected population of sporadic ALS comprises approximately 67% males.1 In familial ALS, an almost 1:1 ratio of males to females is noted (Brown et al. 2017; Al-Chalabi et al. 2016).Diagnosis is primarily determined by clinical examination coupled with nerve conduction studies (NCSs), electromyography (EMG), and laboratory testing (blood count, electrolytes, liver and thyroid function tests, creatine kinase, erythrocyte sedimentation rate, antinuclear antibody, rheumatoid factor, vitamin B12, anti-GM1 ganglioside antibody, serum protein electrophoresis with immunofixation, and 24-h urine protein electrophoresis with immunofixation (Raymond et al. 2018).

The primary initial symptom of ALS is progressive, unilateral weakness of the distal lower extremities and arms without remission or relapse. Atypical symptoms include emotional lability, frontal lobe-type cognitive dysfunction, weight loss, seizures, and cramps without muscle weakness. Patients may also experience other symptoms such as muscle spasms or seizures, trouble swallowing or chewing, and simple tasks such as buttoning up. Unfortunately, ALS patients are aware of a gradual decline in their ability to function. They tend to retain higher mental functions such as problem-solving, reasoning, understanding, and memory (Amyotrophic lateral sclerosis (ALS) fact sheet 2023).

There is currently no effective therapy for ALS and proposed gene or stem cell therapies are still challenging (Rice et al. 2003).An overview of current cell therapies in ALS patients is presented in Table 1. The etiology of neuronal atrophy is unknown, and there is no causal or treatment to slow disease progression. Treatment for ALS is only symptomatic. The course of the disease depends on a person's individuality and the environment in which he lives. Unfortunately, the condition usually progresses to death within 2–4 years. The only drugs that alleviate the course of the disease are riluzole and edaravone. Riluzole reduces glutamate toxicity but prolongs life by only about 3 months, and edaravone has recently been approved, but no survival data have been shown (Surma and KamilMaciąg 2020; Gójska 2008). Cell therapies focus on delaying disease progression and suppressing inflammation. Current research is aimed at selecting the appropriate therapeutic dose of cells.

**Table 1:** Selected clinical trials for ALS

Amyotrophic lateral sclerosis (ALS)				
Year	Animal model	Type of cells	Therapeutic effect	Literature
2009	Human	Human spinal cord-derived NSCs	1. All patients tolerated the treatment without any long-term complications related to either the surgical procedure or the implantation of stem cells 2. One patient has shown improvement in his clinical status 3. Delivery of a cellular payload to the cervical or thoracolumbar spinal cord was well tolerated by the spinal cord in this vulnerable population	Petrou et al. (2021)
2011	Human	Fetal human neural stem cells from natural in utero death (hNSCs)	1. No increase of disease progression due to the treatment was observed for up to18 months after surgery 2. Rather, two patients showed a transitory improvement of the subscore ambulation on the ALS-FRS-R scale	Mazzini et al (2019)
2016	Human	Bone marrow-derived mesenchymal stem cells (BM-MSCs)	1. No results yet	[NCT02881489]
2019	Human	Bone marrow-derived MSCs induced to secrete neurotrophic factor (MSC-NTF)	1. Patients with rapid progression, the rate of disease progression improved at early time points, 2. After 2 weeks, CSF neurotrophic factors increased and CSF inflammatory biomarkers decreased, 3. Single-dose MSC-NTF cell transplantation is safe and shows early promising signs of efficacy 4. In the Phase III trial, significant improvements in cerebrospinal biomarkers of neuroinflammation, neurodegeneration, and neurotrophic factor support were observed with MSC-NTF, with the placebo unchanged	Berry et al. (2019)
2020	Human	Wharton's jelly mesenchymal stem cells (WJ-MSCs)	1. The median survival time for patients increased twofold, 2. Three types of disease progression were observed as measured by the ALSFRS-R: decreased, no change, and increased progression rate. However, the risk-to-benefit ratios were favorable 3.No serious adverse effects of the drug were observed	Barczewska et al. (2020)
2020	Human	Autologous bone marrow-derived mesenchymal stem cells (BM-D MSCs)	1. Patients with a naturally rapid course, a slowing of the disease was observed after treatment, but the group proved to be too small to assess whether the changes were statistically significant	Siwek et al. (2020)
2021	Human	Mesenchymal stem cells (MSC)	1. Patients demonstrated an improvement in the ALSFRS-R progression rate over the entire period to the last transplant 2.Repeated intrathecal injections of autologous MSC were safe in ALS patients and provide clues about the mid-term clinical benefits associated with the cell injection intervals	[NCT04821479]

#### hiPCS in ALS

Neurodegenerative diseases, such as ALS, are characterized by high etiological heterogeneity, with diverse genetic causes, environmental factors, and complex pathophysiologies played out in the multicellular environment of the aging nervous system. This complexity poses significant challenges for in vitro modeling. However, the advent of human induced pluripotent stem cell (hiPSC) technology in 2006 has dramatically changed our ability to create physiologically relevant in vitro models for such diseases. As the genetic background of the cell donor is maintained during cell reprogramming, hiPSCs provide an excellent means to evaluate the effect of disease-causing mutations on the relevant, otherwise non-accessible cell types in neurodegenerative disorders, such as neurons and glial cells. In addition to monoculture differentiation protocols, more complex in vitro models, including multicellular and three-dimensional (3D) culture compositions, are also becoming available to capture disease-relevant cellular interactions (Giacomelli et al. 2022).We have several options for using hiPSC in ALS research:

1. Direct differentiation of hiPSC spinal motor neurons (MN), the first important step towards their application in the study of neurodevelopment, neurodegeneration, and potential future use in the clinic.
2. Differentiation of hiPSCs into astrocytes and microglia. In neurodegenerative disorders such as ALS, astrocytes and microglia are thought to play a role in pathogenesis by promoting inflammation in the brain (neuritis). In the brain and spinal cord, astrocytes and microglia perform their primary functions in neurons and signal each other (Matejuk and Ransohoff 2020). Although changes in these interactions are associated with ALS (Filipi et al. 2020), it remains unclear what triggers nerve inflammation and how these changes contribute to neurodegeneration in ALS. Nevertheless, controlling neural inflammation by modulating MN-glial communication is under active research as a potential therapeutic strategy for ALS.
3. Differentiation of hiPSCs into astrocytes, consisting of four steps (1) differentiation of hiPSCs into neural progenitor cells (NPCs); (2) neural patterning to specify astrocytes to defined regions of the CNS; (3) long-term culture or induction of gliogenic switch; and (4) astrocyte terminal differentiation and maturation (Tyzack et al. 2016).
4. Differentiation of hPSCs are microglia, consisting of four steps (1) initial patterning into cells corresponding to the primitive streak; (2) differentiation into hemangioblasts and primitive hematopoietic stem cells; (3) differentiation into myeloid progenitor cells; (4) terminal microglial differentiation and maturation.

However, modeling with hiPSC has many challenges ahead of it, for example, weak technical reproducibility and lack of modeling inter-cellular crosstalk. Possible solutions include cell purification strategies and developing more complex, multicellular, and three-dimensional culture systems (Giacomelli et al. 2022). iPCS is increasingly used in ALS research. Although the studies published so far have mainly focused on iPSC-based ALS cellular models for disease mechanisms or drug screening (Burkhardt et al. 2013; Lee et al. 2018; Hawrot et al. 2020). Several studies of iPSC-derived NSC transplants using ALS mouse models have shown positive effects of therapy, including life extension (Popescu et al. 2013; Kondo et al. 2014; Nizzardo et al. 2014). However, no clinical trials have translated iPSCs into ALS cases. The advantages of using iPSC in treating neurodegenerative diseases with stem cells include the lack of the need for immunosuppression. Because iPSCs are autologous SCs, there is no ethical issue here. On the other hand, potential disadvantages of iPSCs include that patient-derived iPSCs may develop with similar post-transplant degeneration over time. Recently, successful transplantation of iPSC-derived dopaminergic neurons in patients with Parkinson's disease was reported, with significant improvement in patient's symptoms and quality of life (Schweitzer et al. 2020), opening a new avenue of therapeutic approach not only for Parkinson's disease but also for other neurodegenerative diseases such as ALS (Je et al. 2021).

#### MSCs and NSCs in ALS

The general goal of basic stem cell research in ALS is to replace damaged neurons and reverse their function by preventing further degeneration of weakened neurons and regulating the surrounding niche through the production and release of neurotrophic factors (Kolagar et al. 2020; Terashima et al. 2020; Munter et al. 2020 May). For example, although previous studies have shown the migration of bone marrow-derived cells into the spinal cord of ALS mice and their neuroprotective effects (Terashima et al. 2020), such results were only partial, which may raise the possibility that additional modulation of the spinal cord niche is necessary to optimize neuroprotection. One alternative strategy to improve the efficacy of SC therapy in these models is to generate trophic factors expressing MSCs using human artificial chromosome vectors (Watanabe et al. 2015; Sinenko et al. 2020).

Promising results have been obtained in animal studies regarding using MSCs in ALS. For example, intravenous (IV) and intrathecal (IT) administration of MSCs has been shown to suppress experimental autoimmune encephalomyelitis (EAE) (Kassis et al. 2008; Harris et al. 2012) and promote remyelination after a spinal injury or induced demyelination (Hedayatpour et al. 2013; Zhang et al. 2010).

In a mouse model of ALS, MSC administered intravenously (IV), intrathecally (IT), or intrathecally improved motor performance and prolonged survival of the animals.

Auspicious results of clinical trials were conducted with mesenchymal stem cells induced to secrete high levels of neurotrophic factors (MSC-NTF), a novel autologous cell therapy capable of targeting multiple pathways, which could safely slow ALS disease progression.

Forty-eight patients were administered autologous bone marrow-derived MSCs induced to secrete neurotrophic factor (NTF). They were delivered by simultaneous intrathecal and intramuscular administration to amyotrophic lateral sclerosis (ALS) participants. In the subgroup of subjects with rapid progression, the rate of disease progression improved at early time points. At 2 weeks after cell administration, CSF neurotrophic factors increased, and CSF inflammatory biomarkers decreased. The results show that single-dose MSC-NTF cell transplantation is safe and shows early promising signs of efficacy (Berry et al. 2019). In the Phase III trial, participants received three treatments of MSC-NTF or placebo intrathecally.33% of MSC-NTF and 28% of placebo participants met clinical response criteria at 28 weeks. A pre-specified analysis of participants' response rate at 28 weeks was 35% MSC-NTF and 16% placebo.Significant improvements in cerebrospinal biomarkers of neuroinflammation, neurodegeneration, and neurotrophic factor support were observed with MSC-NTF, with the placebo unchanged.The treatment was well tolerated by patients but did not reach statistical significance. However, it provided important information about the study design and potential biomarkers of treatment response for use in future clinical trials. Patients with the lying form of the disease were able to maintain better function compared to the placebo group (Cudkowicz et al. 2022).

Mazzini et al. conducted a study to evaluate the feasibility and safety of administering a line of human neural stem cells (hNSCs). Eighteen patients with ALS received microinjections of hNSCs into the gray matter pathways of the lumbar or cervical spinal cord. Patients were monitored before and after transplantation with clinical, psychological, neuroradiological, and neurophysiological assessments. A transient decrease in progression (1–4 months after cell administration) on the ALS functional assessment scale was present, and there were no side effects or disease progression. Such a transplant is the first example of a highly standardized cellular therapeutic product. It can be reconstituted and stably expanded ex vivo. Given this and the potential therapeutic effects, further studies using hNSCs are needed (Mazzini et al. 2019).

Another study aimed to evaluate the effect of Wharton's jelly mesenchymal stem cells (WJ-MSCs) on disease progression. The study included 67 ALS patients. Patients were assessed using the ALSFRS-R scale. Three intrathecal injections of WJ-MSCs were administered every 2 months, and the median survival time for patients increased twofold. Regarding disease progression, three types of responses were observed as measured by the ALSFRS-R: decreased no change and increased progression rate. However, the risk-to-benefit ratios were favorable. No serious adverse effects of the drug were observed. This study showed that Wharton's jelly-derived mesenchymal stem cell therapy is safe and effective in some ALS patients (Barczewska et al. 2020). A similar study of intrathecal injection of autologous bone marrow-derived mesenchymal stem cells (BM-D MSCs) was used for 3 administrations over 3 months. Outcomes were also measured using ALSFRS-R. The safety of MSC injections was confirmed, and various effects of the therapy were documented. In patients with ALS with a naturally slow course, there was no significant change in the rate of disease progression. Still, in those with a naturally rapid approach, the disease slowed after treatment with MSCs. Unfortunately, the group proved too small to assess whether the changes were statistically significant. The major limitation of this treatment method is the lengthy preparation of BM -D MSCs cells for transplantation (Siwek et al. 2020).

In phase 2 clinical trial, Petrou et al. evaluated the safety and efficacy of repeated intrathecal administration of autologous MSC in patients with ALS. The study included 20 people (aged 20–70) with a definitive diagnosis of ALS and a score of > 20 on the ALSFRS-R scale (Amyotrophic Lateral Sclerosis). Patients were treated with 1–4 intrathecal MSC injections at 3–6 month intervals. The primary endpoints were safety and tolerability. Efficacy measures were assessed as secondary endpoints, including the ALSFRS-R score and Forced Vital Capacity (FVC). No serious adverse events were observed throughout the study period. The monthly ALSFRS-R progression rate was improved by more than 25% in 15/19 patients between injections 1 and 2 (mean improvement 107.1%), 11/12 between the 2nd and 3rd injections, and 8/10 between the 3rd and 4th injections. Overall, 13 patients had a > 25% improvement in the ALSFRS-R progression rate over the entire period to the last transplant. Seven out of 19 patients had an actual clinical improvement (ALSFRS-R increase range: + 1 to + 4 degrees) after the first transplant, and 5 improved after the second cycle. The speed of response correlated with the time interval between injections. The results of this study show that repeated intrathecal injections of autologous MSC were safe in ALS patients and provide clues about the mid-term clinical benefits associated with the cell injection intervals. More extensive studies are needed to confirm these observations (Petrou et al. 2021).

In 2016 scientists started to investigate the safety and tolerability of autologous bone marrow-derived mesenchymal stem cells (BM-MSCs) administration in patients with diagnosed amyotrophic lateral sclerosis [NCT02881489]. Patients are divided into two groups: Group I—receiving one intrathecally application, and Group II—accepting three applications of BM-MSCs. Subsequently, BM-MSCs will be applied to the cerebrospinal fluid. The effectiveness of the therapy will be assessed by, among other things: the safety of the treatment and the presence of adverse events, and the Functional Rating Scale (FRS) will be monitored to determine the ALS progression rate. However, the results of this study have yet to be discovered as the investigation is still ongoing.

On the other hand, NSC therapy replaces lost cells by preserving the remaining cells with the supporting roles of NSCs (Haidet-Phillips et al. 2011). Implanted human NSCs have been shown to delay disease onset and progression and increase overall survival in mouse models of ALS through multiple actions, including the production of neurotrophic factors and the reduction of neural inflammation (Knippenberg et al. 2017; Teng et al. 2012). Glial cells and NSCs can secrete neurotrophic and anti-inflammatory factors that affect neuroprotection (Haidet-Phillips et al. 2011). Spinal transplantation appears to be most effective for NSC (Teng et al. 2012). Various levels of intrathecal administration have been performed in preclinical and clinical trials of NSC, including the cervical and lumbar levels. The results of these studies are promising and show that after transplantation, human NSCs successfully integrate into the host tissue parenchyma, differentiate into glial cells and neurons, and induce beneficial effects through the release of growth factors (IGF-1, GDNF, etc.) and immunomodulation (Knippenberg et al. 2017; Teng et al. 2012). However, several hurdles still exist, including the length of survival of NSCs in tissue and immune-mediated allograft rejection (Petrou et al. 2021).

In 2009, the FDA approved the first phase 1 clinical trial of transplantation of human spinal cord-derived NSCs into ALS patients, which was subsequently conducted in a phase 2 clinical trial (Glass et al. 2012; Feldman et al. 2014). These studies have proven their safety, but they have failed to slow the progression of the disease with treatment. In 2011 Mazzini et al. approved a phase 1 human fetal NSC clinical trial in Italy, demonstrating its safety and feasibility (Mazzini et al. 2015).Several patients experienced a transient improvement in the first 4 months of this clinical trial with human fetal NSC transplantation.

Based on previous preclinical and clinical studies, more future work will be needed to achieve maximum benefit from stem cell therapy for ALS. In addition, establishing standardized protocols for cell preparation and transplantation will help generate accurate and reproducible data for future preclinical and clinical studies. Combined clinical trials with appropriate modifications, many patients, and long-term follow-ups are necessary to gain important insights regarding stem cell therapy's efficacy, safety, and feasibility in ALS.

### Multiple Sclerosis

Multiple sclerosis (MS) is a chronic neuroinflammatory autoimmune disease of the human central nervous system (CNS). Nearly three million people worldwide suffer from MS (Walton et al. 2020), especially young women (Gbaguidi et al. 2022).

Although the cause of the disease remains unknown, it has become evident that environmental factors and genetic factors increase susceptibility to this disease (Reich et al. 1006; Coyle 2021; Lulu et al. 2016). In addition, viral infections, especially the Epstein-Barr virus, are also included as risk factors to increase the likelihood of developing MS (Venkatesan and Johnson 2014) (32-fold increase in disease susceptibility). In multiple sclerosis, brain-responsive, encephalitogenic T cells (especially TH cells) from the body periphery invade the brain (Kaufmann et al. 2021) and induce a self-destructive immune response that leads to changes in both white and gray matter. These autoreactive T lymphocytes are the main drivers of the disease (Lassmann and Bradl 2017).However, abnormally activated glial cells (Mass et al. 2017; Goldmann et al. 2013) and B lymphocytes (Mass et al. 2019) also play an essential role. Long-term depletion of B cells by targeting CD20 with monoclonal antibodies can attenuate disease progression in relapsing–remitting and primary progressive MS (Hauser et al. 2017; Montalban et al. 2017; Myhr et al. 2019; Schwarz and Schmitz 2023).

MS patients have numerous clinical symptoms that reflect the sites of CNS damage.

Symptoms include:

- Visual disturbances/optic neuritis.
- Central motor paresis.
- Sensory dysfunctions (numbness/paresthesias).
- Sensory ataxia (Ford 2020; Mross et al. 2022; Wilkins 2017).

These clinical signs are "white matter" symptoms resulting from demyelination and axonal damage of the corresponding white matter fibers. The most common form of multiple sclerosis is relapsing–remitting multiple sclerosis (RRMS). The disease is characterized by acute inflammatory episodes that subside to some extent. Over time, symptoms become progressively chronic and worsen without remission or only with incomplete remission (progressive forms of multiple sclerosis) (Lassmann et al. 2012). Gray matter abnormalities in MS patients include memory dysfunction, fatigue, and mood disorders (e.g., depression) (Bellingacci et al. 2021; Filippo et al. 2021). These cortical dysfunctions occur even in the early stages of the disease (Haji et al. 2012), independently of white matter demyelination, and are difficult to reconcile with white matter-only changes.

The therapeutic options for progressive multiple sclerosis are relatively disappointing and remain challenging. One possible reason is the lack of understanding of the pathogenic mechanisms leading to progressive multiple sclerosis. Numerous drugs targeting various pathogenic mechanisms of multiple sclerosis progression are developing. The compounds may target immune system dysfunction (B cells and microglia), glial cells or neurons, metabolic abnormalities associated with mitochondrial damage, or ion channels. In addition, several trials are currently underway using neuroprotective therapies to stop progression or reparative therapies to at least partially reverse some aspects of neurological disability by repairing brain and spinal cord tissues (Correale et al. 2017). Frequently used disease-modifying treatments for MS include ocrelizumab, rituximab, natalizumab, fingolimod, and dimethyl fumarate. Siponimod and orcelizumab treat progressive MS. Many of these agents are still in subsequent phases of clinical trials and are not yet widely used. Therefore, stem cells are increasingly important in treating MS (Hauser and Cree 2020).

A number of clinical trials using hematopoietic stem cells and mesenchymal stem cells have been undertaken, and are shown in Table 2. The therapy has proven to be safe for patients and decrease in ALS progression. Promising results have come from studies using adipose stem cells and nanovesicles (ASC-NVs) in a rat model, but further studies are needed to use the therapy in human treatment.

**Table 2:** Selected clinical trials for MS

Multiple sclerosis (MS)				
Year	Animal model	Type of cells	Therapeutic effect	Literature
2010	Human	MSCs	1. MRI showed MSC cells in the occipital horns of the ventricles, indicating possible migration of ferum-labeled cells in the meninges, the subarachnoid space, and the spinal cord 2. 72% of patients increased the percentage of regulatory T cells (CD4 + CD25 +), decreased proliferative responses of lymphocytes, and the expression of CD40 + , CD83 + , CD86 + , and HLA-DR on myeloid dendritic cells	Karussis et al. (2010)
2011	Human	Hematopoietic stem cells (HSC)	1. PFS rate was significantly better in patients with active MRI lesions, 2. Significant reduction in the number and volume of gadolinium-enhancing lesions on MRI	Fassas et al. (2011)
2012	Human	Hematopoietic stem cells (HSC)	1. The overall clinical response in terms of disease improvement or stabilization was 80%, 2. No active, new, or enlarging lesions in magnetic resonance imaging were registered in patients without disease progression	Shevchenko et al. (2012)
2012	Human	Mesenchymal stem cell (MSC)	1. MSC treatment was safe and well tolerated 2. It show no effect on GEL, an MRI surrogate marker of acute inflammation, in patients with active multiple sclerosis at week 24	Uccelli et al. (2021)
2014	Human	Umbilical cord MSC (UCMSC)	1. The improvement of the patient's condition was most visible 1 month after treatment. Improvements were seen in EDSS scores 2.Treatment with UCMSC intravenous infusions for patients with MS is safe, and potential therapeutic benefits should be further investigated	[NCT02034188]
2014	Human	Mesenchymal stem cell (MSC)	1. At 1-year follow-up, 58.6% and 40.6% of patients treated with MSC-IT and MSC-IV showed no signs of disease activity 2. Transplantation produced additional benefits in terms of relapse rate, monthly changes in T2 lesion load on MRI, 25-foot walk test, 9-hole peg test, optical coherence tomography, functional MRI, and cognitive testing 3. Intrathecal administration was more effective than intravenous in several disease parameters	[NCT02166021]
2018	Rats	Adipose Stem Cells + nanovesicles (ASC-NVs)	1. Reduction of the severity of experimental autoimmune encephalomyelitis (EAE), 2. Reduction of inflammation and demyelination in the spinal cord, 3. Reduced activity among CNS immune cells, including decreased microglia and T-cell extroversion	Farinazzo et al. (2018)
2019	Human	Hematopoietic stem cell (HSC)	1. Prolonged time to disease progression, 2. The mean EDSS scores decreased (improved) during the first year	[NCT00273364], Burt et al. (2019)
2021	Human	Autologous neural progenitors derived from mesenchymal stem cells (MSC-NP)	1. CSF biomarkers altered in response to MSC-NP treatment may reflect specific immunoregulatory and trophic mechanisms of therapeutic response in MS	Harris et al. (2020)
2022	Human	Hematopoieticstemcells (HSC)	2. The higher baseline frequencies of specific pro-inflammatory immune cells, increased frequencies and absolute counts of immunoregulatory CD56hi natural killer cells, and terminally differentiated CD8 + CD28- CD57 + cells	Visweswaran et al. (2022)
2022	Human	Mesenchymalstemcells (MSCs)	1. Levels of NF-L were significantly lower at 6 months after treatment	Petrou et al. (2022)

Therapeutic solution is supporting the mobilization of stem cells. This effect can be achieved by administering various trophic and growth factors, such as EPO (erythropoietin) (Tsiftsoglou 2021), BDNF (brain-derived neurotrophic factor) (Xia et al. 2021) or GDNF (glial-derived neurotrophic factor) (Chiavellini et al. 2022), chemokines, such as SDF-1 (stromal cell-derived factor 1) cytokines (Jiang et al. 2022).

In Karussiset al. (2010) have conducted studies with MSC in both MS and ALS patients. All stem cells were obtained by intrathecal injection in the lumbar region. Some patients have also received treatment with MSCs intravenously. MRI showed MSC cells in the occipital horns of the ventricles, indicating possible migration of forum -labeled cells in the meninges, the subarachnoid space, and the spinal cord. After 24 h after transplantation, the analysis of data for all patients in total showed a 72% increase in the percentage of regulatory T cells (CD4+ CD25+), decreased proliferative responses of lymphocytes, and the expression of CD40+ , CD83+ , CD86+ and HLA-DR on myeloid dendritic cells. Additionally, after the stimulation of lymphocytes with phytohaemagglutinin, there was a 63% decrease in the number of proliferative cells in the immune response (DimitriosKarussis et al. 2010).

Riordano et al. conducted a clinical trial to test the efficacy and safety of umbilical cord MSC (UCMSC) administration in treating MS (20 subjects). In this one-year study, consenting subjects received seven intravenous infusions of 20 × 106 UCMSC over 7 days. Efficacy assessed at baseline, 1 month, and 1-year post-treatment, including Magnetic Resonance Imaging (MRI), Kurtzke's Expanded Disability Status Scale (EDSS), Scripps Neurological Rating Scale, Nine-hole Test, 25-foot Walk Test, and RAND Short Form-36 quality-of-life questionnaire. Nosevere adverse reactions have been reported. The improvement of the patient's condition was most visible 1 month after treatment. These results may suggest that treatment with UCMSC infusions in patients with multiple sclerosis is safe, and potential therapeutic benefits should be further investigated (Riordan et al. 2018).

In turn, Petrou et al., in a clinical study (trial registration: NCT02166021), evaluated the optimal method of administration, safety, and clinical effectiveness of mesenchymal stem cell (MSC) transplantation in patients with active and progressive multiple sclerosis. Forty-eight patients (28 men and 20 women) with advanced evidence of clinical worsening or activity during the previous year. Patients were randomized into three groups and treated intrathecally (IT) or intravenously (IV) with autologous MSCs (1 × 106/kg) or sham injections. After 6 months, half of the patients in the MSC-IT and MSC-IV groups were re-challenged with MSC and the other half with sham injections. Patients initially assigned to sham treatment were divided into two subgroups and treated with either MSC-IT or MSC-IV. The duration of the study was 14 months. No serious safety concerns have been identified. Significantly fewer patients experienced treatment failure in the MSC-IT and MSC-IV groups than in the sham group. At 1-year follow-up, 58.6 and 40.6% of patients treated with MSC-IT and MSC-IV showed no signs of disease activity compared to 9.7% in the sham group. MSC-IT transplantation produced additional benefits regarding relapse rate, monthly changes in T2 lesion load on MRI, 25-foot walk test, 9-hole peg test, optical coherence tomography, functional MRI, and cognitive testing. Treatment with MSCs was well tolerated in progressive multiple sclerosis and produced short-term beneficial effects on significant endpoints, especially in patients with active disease. Intrathecal administration was more effective than intravenous in several disease parameters. However, it was a phase II study, and continuing it in subsequent clinical trials is necessary. However, the results are promising, as with the rest of the MSC studies (Petrou et al. 2020).

The critical study, as it highlights some of the problems associated with using MSCs, was done by Ucelli et al. It was a phase 2 randomized trial, the great advantage of which was that it was conducted in 15 centers in nine countries. Its purpose was to investigate the safety and activity of MSC treatment. Patients (18–50 years of age) with active relapsing–remitting or progressive multiple sclerosis were enrolled if their disease had lasted 2–15 years from the onset of multiple sclerosis and had an Expanded Disability Scale score of 2.5–6.5. Patients were randomized (1:1) in a crossover fashion to receive a single intravenous dose of autologous bone marrow-derived MSCs and a placebo at the 24th Week 48 follow-up visit. The primary safety endpoint was the number and severity of adverse events in each treatment arm. The primary efficacy endpoint was the number of gadolinium-enhancing changes (GEL) counted at weeks 4, 12, and 24 for a between-treatment comparison. The primary efficacy endpoint was assessed in the full set of analyzes after all subjects had completed the week 24 visit. Efficacy endpoints were evaluated using a predefined statistical testing procedure. Safety was monitored throughout the study, with vital signs and adverse events recorded at each visit. Results From July 16, 2012, to July 31, 2019, 144 patients were randomized to the first early intravenous infusion of autologous bone marrow-derived MSCs (*n* = 69) or placebo (*n* = 75). MSC treatment failed to meet the primary efficacy endpoint of total GEL cumulative from baseline to week 24. Two hundred thirteen adverse events were recorded, similarly distributed between the groups. The most commonly reported adverse events were infections and infestations. All serious adverse events were considered not related to the medicinal infusion. No deaths were reported during the study. Based on the results, bone marrow-derived MSC treatment was safe and well tolerated. Still, it showed no effect on GEL, an MRI surrogate marker of acute inflammation, in patients with active multiple sclerosis at week 24. Thus, this study does not support using bone marrow-derived MSCs in treating active multiple sclerosis (Uccelli et al. 2021).

Further research should address the impact of MSCs on parameters related to tissue repair. This study shows that the use of MSCs in MS has its limitations. Its advantage is the large number of patients compared to other studies of this type and the countries in which it was carried out. Which certainly adds to its credibility (Uccelli et al. 2021).

In most of the studies conducted, the effectiveness of auto-HSCT was defined as progression-free survival (PFS). Clinical and MRI outcomes of 35 patients with aggressive MS treated with HSCT. After 15 years of disease, progression-free survival (PFS) is 44% for patients with active CNS disease. PFS rate was significantly better in patients with active MRI lesions, and HSCT also considerably reduced the number and volume of gadolinium-enhancing lesions on MRI. Unfortunately, two patients died from transplant-related complications (Fassas et al. 2011) in 95 patients with different types of MS. After HSCT, efficacy was evaluated based on clinical and quality-of-life outcomes. At long-term follow-up (about 4 years), the overall clinical response regarding disease improvement or stabilization was 80%. No active, new, or enlarging lesions in magnetic resonance imaging were registered in patients without disease progression (Shevchenko et al. 2012).

In 2018 Farinazzo demonstrated in a mouse model that preemptive intravenous administration of nanovesicles (NVs) isolated from adipose stem cells (ASC-NVs) before disease onset significantly reduces the severity of experimental autoimmune encephalomyelitis (EAE) and reduces inflammation and demyelination in the spinal cord. Therapy reduced activity among CNS immune cells, including decreased microglia and T-cell extroversion (Farinazzo et al. 2018).

In a preliminary study, [NCT00273364] of 110 patients with relapsing–remitting MS, hematopoietic stem cell therapy (HSCT) resulted in a prolonged time to disease progression. Patients were randomized to receive HSCT along with cyclophosphamide and anti-thymocyte globulin. Disease progression occurred only in 3 patients in the HSCT group. During the first year, mean EDSS scores decreased (improved). There were no deaths and no potential life-threatening events (Burt et al. 2019).

A study of patients with progressive multiple sclerosis was conducted to determine the long-term safety and efficacy of administering autologous neural progenitors derived from mesenchymal stem cells (MSC-NP). The safety and efficacy of intrathecal MSC-NP treatment were maintained for 2 years, but the degree of disability reversal was not fully supported. CSF biomarkers altered in response to IT-MSC-NP treatment may reflect specific immunoregulatory and trophic mechanisms of therapeutic response in MS (Harris et al. 2020).

To check the longer-term immune reconstitution affects autologous hematopoietic stem cell transplantation (AHSCT) after 2 and 3 years post-transplant, high-dimensional immunophenotyping of peripheral blood mononuclear cells from 22 MS patients was performed using flow cytometry panels. Results have shown that AHSCT induces significant recalibration of pro-inflammatory and immunoregulatory components of the immune system of MS patients for up to 3 years after therapy. The higher baseline frequencies of specific pro-inflammatory immune cells (T-helper-17 (Th17) cells), increase in frequencies and absolute counts of immunoregulatory CD56 + natural killer cells, and terminally differentiated CD8+ CD28—CD57 + cells were observed (Visweswaran et al. 2022).

In another study, possible neuroprotective effects of mesenchymal stem cell (MSC) transplantation (intrathecal or intravenous) in patients with MS were observed. The CSF samples were obtained from 48 patients with progressive MS. The observations were conducted before the first injection and after 6 months of treatment. Neurofilament light chains (NF-L) were shown to serve as a reliable biomarker of neurodegeneration in MS. The CSF levels of NF-L were significantly lower 6 months after treatment with intrathecal MSC compared with pre treatment measurements (Petrou et al. 2022).

As with amyotrophic lateral sclerosis, induced pluripotent stem cells (iPSCs) are also being tested as a treatment for MS. Remarkable improvements in mobility and strong remyelination was observed after transplantation iPSC-derived nerve cells into demyelinated models. Direct reprogramming somatic cells into induced neural cells, such as induced neural stem cells (iNSCs) and induced oligodendrocyte progenitor cells (iOPCs), without going through the pluripotency stage, is an alternative to transplantation that is effective in the congenital hypomyelination model. iPSC technology is advancing as efforts are being made to increase the effectiveness of iPSC therapy and reduce its potential side effects. It gives hope that they will become an alternative to using MSCs in treating multiple sclerosis (Xie et al. 2016).

Numerous clinical trials on using stem cells to treat MS have been or are still being carried out. It is only possible to present some of them. However, after analyzing most of the current research, MSC injections are most often considered for MS. The effectiveness of their use is usually satisfactory. Still, not all studies have obtained the so-called endpoints. Most researchers indicate the need to conduct subsequent phases of clinical trials, which should be based on checking the safety and effectiveness of MSCs.

### Parkinson's Disease

Parkinson's disease (PD) is one of the most common neurodegenerative diseases, mainly affecting people over 50. PD is the second most common neurodegenerative disease that has weakened 1% of the population over the past 60 years and is a particular problem in aging societies (Kopen et al. 1999). Currently, we can distinguish at least three main types of this disease. In the first type—sporadic, in which the cause of the disease is unknown, we can only assume a nucleotide change in some genes, but their direct effect is not specified. The second type—environmental, is the effect of chemical poisons known for years, which specifically destroy dopaminergic neurons. The third type—familial Parkinson's disease, is caused by a specific mutation in one of the genes: alpha-synuclein, parkin, ubiquitin L1 hydrolase, and PINK1 kinase. The protein kinase PINK1 is involved in the control of the state of the mitochondria. It manifests itself in a constantly progressive disturbance of motor functions. The clinical symptoms of PD include increased muscle tension of the plastic type, resting tremors, slow movement, or posture disorders. Physiotherapeutic procedures are carried out to improve the body's overall efficiency and gait pattern, strengthen core muscles, and improve central stabilization. Physiotherapeutic methods used in working with PD patients prevent permanent disability and help maintain independence in everyday life. Still, despite rehabilitation and pharmacological treatment, the patient's ability to function daily is reduced, leading to a significant deterioration in the quality of life (Friedenstein et al. 1970). When the cells were administered, there were motor improvements in the animals. Therefore it was decided to continue human studies. The results are shown in Table 3. Studies are currently underway to determine the appropriate therapeutic dose.

**Table 3:** Selected clinical trials for PD

Parkinson’s disease (PD)				
Year	Animal model	Type of cells	Therapeutic effect	Literature
2008	Sprague–Dawley(SD) rats	Adipose-Derived Adult Stromal cells (ADAS) and human mesenchymal stem cells (hMSCs)	1. Reduction of the degeneration of the nigrostriatal pathway and the behavioral deficits induced by 6-OHDA 2. They attenuate microglia activation 3. Express neurotrophic factors that protect dopaminergic neurons and promote their survival	McCoy et al. (2008); Park et al. (2008)
2012	Femaleathymicrats	Human amniotic fluid stem cells (hAFSCs) and bone marrow-derived mesenchymal stem cells (hBMSCs)	1. Improvement of bladder capacity, micturition pressure, spontaneous activity, and threshold pressure	Soler and Fullhase (2012)
2013	(MPTP)-lesioned hemi-parkinsonian rhesus monkeys	Neuronal-primed ASCs derived from rhesus monkey (rASCs)	1. The differentiated cells transcribed and expressed various genes specific for dopaminergic neurons, 2. They showed the differentiation of neurons, restoring neuroprotective functions, 3.They showed a better neuroprotective effect than gene therapy alone	Yan et al. (2013)
2014	[(Thy1) -h [A30P] aS] Mouse model	Bone marrow-derived MSCs (BM-MSCs)	1. MSCs expressing eGFP in the OB, cortex, amygdala, striatum, hippocampus, cerebellum, and brainstem, 2. The most significant distribution was within the OB and brainstem, 3. INA showed anti-inflammatory and neuroprotective effects	Danielyan et al. (2014)
2014	Human	Human fetal neural stem cells (h-NSCs)	1. The transplanted dopamine neurons showed a healthy and nonatrophied morphology at all time points 2. Labeling of the mitochondrial outer membrane protein Tom20 and α-synuclein showed a typical cellular pathology in the patients' own substantia nigra, which was not observed in transplanted dopamine neurons 3. These results show that the vast majority of transplanted neurons remain healthy for the long term in PD patients, consistent with clinical findings that fetal dopamine neuron transplants maintain function for up to 15–18 years in patients	Hallett et al. (2014)
2017	Monkey *(Macacafascicularis)*	Pluripotent stem cells (iPSCs)	1. MHC matching was effective in reducing the immune response by inhibiting the accumulation of microglia and lymphocytes in grafts, 2. Increased cell survival in an iPSC graft, 3. MHC alignment increases the survival of the transplanted dopamine neurons	Morizane et al. (2017)
2017	Monkey *(Macacafascicularis)*	Human pluripotent stem cells (iPSCs)	1. Increased dopaminergic neurons of the macaque midbrain, 2. Histological studies have shown that mature dopaminergic neurons extend dense neurites into the host's striatum, 3. Increase in the spontaneous movement, 4. iPSCs did not form any tumors in the brain for at least two years	Kikuchi et al. (2017)
2017	Rats	MSC exosome enriched with miR-17–92 cluster	1. Enhances neuronal plasticity and functional recovery, 2.Stimulation of oligodendrogenesis	Xin et al. (2017)
2019	Human	Neural progenitor cells (NPCs)	1. Motor improvement, 2. Most patients has a better response to the drugs after one year of treatment, 3. The dopaminergic activity of the midbrain has been increased	Madrazo et al. (2019)
2019	Human	Mesenchymal stem cell (MSC)	1. There was a statistically significant decrease in the severity of motor and nonmotor symptoms in the study group in the post-transplant period	[NCT04146519]
2020	Human	Autologous adipose-derived stromal vascular fraction (SVF)	1. Patients improved motor performance with lower levels of dopaminergic treatment after SVF	Carstens et al. (2020)
2020 2022	Human	Human Amniotic Epithelial Stem Cells (hAESCs)	1. No results yet	[NCT04414813] [NCT05435755]
2021	Human	Umbilical cord MSC (UC-MSCs)	1. No results yet	[NCT03550183]

Many current PD therapies only deal with symptoms, not the neurodegeneration underlying Parkinson's disease. Several medications can help manage the symptoms of Parkinson's disease. These include levodopa, dopamine agonists, MAO-B inhibitors, and COMT inhibitors. Physical therapy can help improve the mobility and balance of people with Parkinson's disease. It can include exercises to improve muscle strength and coordination and stretching and flexibility exercises.

To better understand the pathophysiological state, scientists are still looking for models that most accurately reflect the phenotypic symptoms of the disease. Advances in personalized medicine and cell reprogramming are allowing access to previously unattainable cell therapies for a specific patient. These therapies use induced adipose-derived adult stromal cells (ADAS) (McCoy et al. 2008) and human mesenchymal stem cells (hMSCs) (Park et al. 2008). Both of them were administered to rat black matter. The therapy reduced the degeneration of the nigrostriatal pathway and the behavioral deficits induced by 6-OHDA. In addition, it weakened microglia activation and increased the production of neurotrophic factors that protect dopaminergic neurons and promote their survival. ADAS cells have survived after transplantation but do not differentiate into dopaminergic neurons.

#### Stem Cells Treatment

The effect of human amniotic fluid stem cells and bone marrow-derived mesenchymal stromal cells was also assessed in a rat model of Parkinson's disease. Human amniotic liquid stem cells (hAFSCs) and bone marrow-derived mesenchymal stem cells (hBMSCs) were injected into the bladder site of damage. Fourteen days after cell administration, bladder capacity, micturition pressure, spontaneous activity, and threshold pressure were improved (Soler and Fullhase 2012).

The delivery of bone marrow-derived MSCs (BM-MSCs) to the brain was investigated in a transgenic mouse model PD [(Thy1) -h [A30P] aS] by intranasal administration (INA). Seven days after administration, MSCs expressing eGFP in the olfactory bulb (ESR), cortex, amygdala, striatum, hippocampus, cerebellum, and brainstem were documented. The most significant distribution was within the ESR and Brainstem. Moreover, the intranasal examination showed anti-inflammatory and neuroprotective effects of the administered cells (Danielyan et al. 2014).

Neuronal-primed ASCs derived from rhesus monkey (rASCs) combined with adenovirus containing NTN and tyrosine hydroxylase (TH) (Ad-NTN-TH) were implanted into the striatum and substantia nigra of the PD monkey model. The differentiated cells transcribed and expressed various genes specific to dopaminergic neurons. They showed the differentiation of neurons, restoring neuroprotective functions. They showed a better neuroprotective effect than gene therapy alone (Yan et al. 2013).

A major histocompatibility complex (MHC) study was performed using MHC-derived pluripotent stem cells (iPSCs). Immunohistological analyses showed that MHC matching effectively reduced the immune response by inhibiting the accumulation of microglia and lymphocytes in grafts and also increasing cell survival in an iPSC graft. Consequently, MHC alignment increases the survival of the transplanted dopamine neurons (Morizane et al. 2017). A similar study was also performed using human iPSCs. After transplantation, they acted as dopaminergic neurons of the macaque midbrain. Histological studies have shown that mature dopaminergic neurons. extend dense neurites into the host's striatum. An increase in the spontaneous movement of the monkeys after transplantation was also observed. Moreover, the cells did not form tumors in the brain for at least two years (Kikuchi et al. 2017).

In 2017, Shin et al. found that both groups of rats treated with MSC exosomes showed a significant improvement in functional recovery compared to liposome treatment. In addition, treatment with MSC exosome enriched with miR-17–92 cluster had a much stronger effect on improving neurogenesis (Xin et al. 2017), resulting in stimulation of oligodendrogenesis and improved neuronal function.

Human neural progenitor cells (NPCs) were tested in 8 patients with moderate PD. Using modern surgery, NPC suspensions were injected bilaterally into the dorsal putamina of the patients. One year after the cell transplant, patients showed varying degrees of motor improvement, and five showed a better response to the drugs. The dopaminergic activity of the midbrain has been increased. After 4 years of treatment, the progress slightly decreased but remained better than initially. Increased dopaminergic neurotransmission in the dorsal putamina has also been noted, which may correlate with improved motor function (Madrazo et al. 2019).

Recently, two PD patients were treated with an autologous adipose-derived stromal vascular fraction (SVF). They were given directly to the muscles of the face and nose. Both patients showed improved motor performance with lower levels of dopaminergic treatment after SVF. Despite the unknown mechanism of action, this potential therapy requires further research (Carstens et al. 2020).

#### Clinical Trials

There are many clinical trials for Parkinson's disease. While many of these are still ongoing, there have already been initial reports of the effectiveness of stem cell therapy in PD patients.

The h-NSC is a cell therapy consisting of human fetal neural stem cells (h-NSCs). The h-NSC injection has been delivered by nasal route for PD patients.To assess the long -term health and function of transplanted dopamine neurons in patients with Parkinson's disease, the expression of dopamine transporters (DATs) and the morphology of the mitochondria in the cells were examined in human fetal midbrain transplants. DAT was successfully localized in transplanted dopamine neuron terminals in the re-innervated host putamen.The transplanted dopamine neurons showed a healthy and nonatrophied morphology at all time points. Labeling the mitochondrial outer membrane protein Tom20 and α-synuclein showed a typical cellular pathology in the patient's substantia nigra, which was not observed in transplanted dopamine neurons. These results indicate that most transplanted neurons remain healthy long-term in PD patients, consistent with clinical findings that fetal dopamine neuron transplants maintain function for up to 15–18 years in patients.These findings demonstrate that the majority of transplanted neurons are healthy for an extended period in PD patients, which is in line with clinical observations that fetal dopamine neuron transplants have a long-term effect in patients that is consistent with clinical findings that fetal dopamine neurons have a long-term impact on patients (Hallett et al. 2014).

In 2021, a phase I clinical trial was completed [NCT03550183] where researchers used umbilical cord MSC (UC-MSCs) via intravenous infusion to treat PD. With varying durations of follow-up, the researchers tested the therapeutic effect and quality of life. Unfortunately, there are no results yet. This study [NCT04146519] aimed to assess the immediate consequences of introducing MSCs on the effectiveness of motor and nonmotor symptoms in patients with PD. MSCs were transplanted to 12 patients with PD through intravenous and tandem (intranasal + intravenous) injections. The effectiveness of the therapy was evaluated 1 and 3 months post-transplantation. There was a statistically significant decrease in motor and non motor symptoms in the study group in the post-transplant period. MSCs transplantation is a disease-modifying therapeutic strategy in PD (Boika et al. 2020).

In 2020 [NCT04414813] and 2022 [NCT05435755], clinical trials began in patients with Parkinson's disease. The therapy will be based on Human Amniotic Epithelial Stem Cells (hAESCs), directly injected into the lateral ventricles using modern surgical robots. This study will enable the development of an optimal stem cell treatment strategy.

The future of stem cell therapy for Parkinson's disease looks promising, with ongoing research and clinical trials exploring this innovative approach's potential to treat this debilitating neurological disorder. Here are some potential developments and advancements we may see in the future of Parkinson's stem cell therapy: improved delivery methods, personalized treatments, safer stem cell sources, and combination therapies. Stem cell therapy for Parkinson's disease is a promising area of research, but it also carries potential risks and dangers. Here are some of the main dangers associated with stem cell therapy for Parkinson's disease: tumor formation, immune rejection, and side effects: such as fever, headaches, and infections. It's important to note that these dangers are not unique to stem cell therapy for Parkinson's disease. They are potential risks associated with any stem cell therapy. However, researchers are working to address these risks and make stem cell therapy safer and more effective for patients with Parkinson's disease.

### Huntington's Disease

Huntington's disease (HD) is a fatal, progressive neurodegenerative disorder. Moreover, it is an autosomal dominant disease. The mechanism behind the condition is that there is an excess of CAG triplets in the huntingtin (HTT) gene, which are converted to polyglutamine (polyQ) residues at the end of the huntingtin (HTT) protein, changing the properties of the protein. When the number of CAG repeats exceeds 40, huntingtin aggregates in the nuclei of neurons, thereby inhibiting the action of various proteins, including key transcription factors. These are pathological changes leading to impaired transcription and degeneration of neurons—the more of these changes, the more advanced the disease phenotype. The presence of chorea diagnoses the clinical manifestations of the disease.The prevalence of Huntington's disease in Western populations is 10.6–13.7 per 100,000. In Japan, Taiwan, and Hong Kong, the majority of HD is 1–7 per million. In South Africa, lower rates are seen in black populations compared to white and mixed people. The difference in the prevalence of the disease in different ethnic groups relates to genetic differences in the HTT gene. High-prevalence populations have longer mean CAG repeats. For example, people of European descent ordinary 18.4–18.7, while those of Asian descent average 16.9–17.45 (McColgan and Tabrizi 2018 Jan). Movement disorders in HD can be divided into a hyperkinetic phase with pronounced chorea into the early stages of the disease, which then tends to plateau. The hypokinetic phase is characterized by bradykinesia, dystonia, and balance and gait disturbances. Hypokinetic movement disorder is associated with disease duration and CAG length, whereas chorea is not. Cognitive impairment in HD can be observed years before symptom onset and is subcortical, characterized by impaired emotion recognition, processing speed, and visuospatial and executive functions (Papoutsi et al. 2014). HD has neuropsychiatric symptoms, including apathy, anxiety, irritability, depression, obsessive-compulsive behavior, and psychosis.

Huntington's disease has a profound impact on quality of life. A decrease in total functional capacity is observed after the symptoms' onset, job loss, and the need to change jobs in the early period. As the disease progresses to the end stage, round-the-clock care is required. Deterioration of motor and cognitive functions is why patients are placed in care institutions (Meer et al. 2012).Pharmacotherapy for HD patients remains insufficient despite increased disorder diagnosis, better genetic counseling, and greater access to specialized care programs. Currently, only palliative therapies that treat the symptoms of the disease (chorea and depression) are approved. The most commonly used drugs are antipsychotics and antiepileptics (Wu et al. 2019).

#### Sources of Stem Cells in HD

Stem cell therapy is an alternative treatment method assuming that new neurons can replace the degenerating cells in the affected brain areas and alleviate the course of the disease (Connor 2018). Trials to date have been undertaken on mouse models, a selection of which is in Table 4, with promising results. A clinical trial is currently underway. Cellavit et al.noted that stem cell therapies with neural progenitor cells (NPCs) derived from induced pluripotent stem cells (iPSCs) have significant potential in the treatment of neurodegenerative diseases, including HD (Wenceslau et al. 2017; Kim et al. 2021a, b, c).NSCs derived from the fetal or adult brain are considered an attractive source of cell therapy for HD. Originally cultured NSCs, fetal-derived immortalized NSC lines, and CNS brain tissue NSCs have been used for transplantation studies in HD animal models. In animal model studies, HD NSCs were transplanted directly into the striatum, showing survival and distribution in injured brain areas. In addition, transplanted NSCs improved both motor and psychological symptoms (Choi and Hong 2017; Barker et al. 2013) but showed limited migration in the transplanted tissues. Moreover, NSCs derived from aborted fetuses generally offer a limited number of cells. However, they may have a chance of tumor growth from residual mitogenic NSCs with a population doubling time of more than 70, which is one of the significant challenges in NSC cell transplant therapy (Casarosa et al. 2014). However, an alternative living source, such as expandable NSCs grown in an in vitro cell culture system, is required.

**Table 4:** Selected clinical trials for HD

Huntington’s disease (HD)				
Year	Animal model	Type of cells	Therapeutic effect	Literature
2010	YAC128 mouse model	Bone marrow MSCs	1. Motor improvements 2. Reduced neuronal loss	Eskandari et al. 2021)
2013	R6/2 mouse model	MSCs	1. Reduced loss of medium spiny neurons 2. Reduced striatal atrophy 3. Reduced Htt aggregation and stimulation of endogenous neurogenesis	Kim et al. (2021a, b, c)
2017	YAC128 mouse model	Induced pluripotent stem cell-derived neural stem cells (iPS-NSCs)	1. Reduced neuronal loss 2. Increased BDNF and tropomyosin receptor kinase (BTrkB) levels in the striatum 3. Improved the mice motility	Fink et al. (2013)
2017	R6/2 mouse model	Adipose tissue-derived MSCs (ADMSCs)	1. Reduces mHtt aggregates in mouse neuronal cells 2. Reduction in abnormal apoptotic protein levels	Al-Gharaibeh et al. (2017)
2018	1. R6/2 2. Q140 HD knock-mouse model	hNSCs	1. In R6/2 mice, integration with existing neuronal circuitry, inhibit synaptic changes and improvement of motility 2. In Q140 knock-in mice, improvement of cognitive impairment in late-stage disease 3. In both models, increased BDNF expression	Lee et al. (2017)
2016	Human	Stem cell therapy- Cellavita HD	1. No results yet	[NCT032535, NCT02728115; NCT04219241]

A common drawback of most approaches that have used NSCs to date is that they appear to have limited ability to differentiate fully functional, pre-synaptic, or post-synaptic competent neurons. Further research must establish new methods for the in vitro differentiation of functional MSNs expressing different reliable markers. Future research should also address important issues regarding the efficacy and safety of pluripotent cell-derived and/or induced NSCs for clinical use. It may lead to tremendous success in stem cell-based HD therapies.

In animal studies, iPSC-derived human neural progenitor cells have been found to implant in the brain and differentiate into normal neuronal cells, promoting behavi oral and motor regeneration (Mu et al. 2014).

Since somatic cells can be transformed into pluripotent stem cells by expressing specific transcription factors, somatic cell reprogramming can be used for personalized cell therapies.iPSCs have been successfully formed from various types of somatic cells, including fibroblasts, blood cells, renal epithelial cells, and keratinocytes. Currently, the best characterized HD iPSC lines have been produced from patient fibroblasts using lentivirus or retrovirus to express a combination of pluripotency factors, including Oct3/4, Klf-4, Sox2, c-Myc, SSEA4, LIN-28, NANOG and p53 shRNA (to increase efficiency) (Tousley and Kegel-Gleason 2016).

Reprogramming mouse and human somatic cells into iPSCs may help understand the cause of the disease and its cure. To generate functional therapeutic cells, hPSCs must be successfully differentiated into the desired cell type. However, current hPSC differentiation protocols typically result in heterogeneous populations of differentiated and undifferentiated cells. Methods to generate a homogeneous population of cells should be developed. One of the significant concerns with PSCs and their derivatives in transplantation therapies is the risk of cancer due to residual undifferentiated cells (Fong et al. 2010). The solution to this problem may be the technology of direct conversion of somatic cells into neuronal cells because neuronal cells are post-mitotic cells.

Direct conversion of somatic cells bypassing the pluripotency state is a powerful tool in cell therapy due to its ability to generate desired cells quickly. Fibroblast reprogramming has recently been reported to be directly transformed into other somatic cell types, such as neuronal cells (Zhang et al. 2014). Generating neuronal cells from somatic cells using neuron-specific transcription factors has shown that generating desired cells from different lineages is possible.

There are numerous iPSC lines with CAG repeat ranging from wild-type to HD in the Huntingtin (HTT) gene (from 17 to 180 CAG). Many of these cell lines were created by the HD iPSC consortium and are available through the newly established NINDS Human Cell and Data Repository (NHCPR), including 8 unbroken and 18 HD iPCS lines (the cell line catalog can be viewed at the following website: https://stemcells.nindsgenetics.org/) These cell lines together are beneficial as they provide an isogenic background against which to determine the effects of the HD mutation. Although isogenic lines are the "gold standard" for a well-controlled iPSC experiment, the inherent variability found among control iPSCs justifies the need for more isogenic lines from additional HD iPSCs (Guo et al. 2013; Camnasio et al. 2012; Ring et al. 2015) .iPSCs from juvenile-onset HD (> 60 CAG) have been used more frequently for genomic and proteomic studies than iPSCs from an adult-onset HD (39–60 CAG) (Mattis et al. 2015). However, only 5% of HD patients have the juvenile disease (before age 20) with associated CAG repeat length greater than 60 (Fink et al. 2015; Quarrell et al. 2012).

Future work with iPSC-derived neural cells may provide insight into the molecular changes underlying the behavioral changes identified in patients with medium repeat lengths (Killoran et al. 2013) and differences in disease penetrance at low repeat lengths.

Considerable progress has been made in HD iPSC, and despite the significant improvement in this field, some problems can be seen with the use of iPCS. The limited number of studies of iPSCs and their differentiated progeny using adult-onset CAG lengths is of concern, as adult-onset HD accounts for the vast majority of HD cases. Further stem cell lines are also needed, as existing stem cell lines age with a passage. New methods using 3D culture or co-breeding systems could be the key to revealing phenotypes without stress. Standardizing the study protocol to improve the comparison between studies is also necessary. Thanks to the pioneering work of many HD researchers, HD iPSCs are just beginning to show promise and may hold the key to finally identifying treatments beneficial for HD patients.

#### Animals Models and Clinical Trials

A significant study in a mouse model was carried out by Jaisan et al. They wanted to see if transplantation of hESCs labeled with superparamagnetic iron oxide (SPION) nanoparticles could migrate in the degenerated neural region and improve motor dysfunction in a Huntington rat model transfected with AAV2-Htt171-82Q. For this purpose, all animals were first randomized into three groups: the HD group, the sham group, and the control group. After 6 weeks, the HD and sham groups' animals were again divided into two subgroups depending on the animals that received the ipsilateral or contralateral hESC transplant. They performed cylinder and step tests every 2 weeks after AAV2—Htt171-82Q injection and hESC transplantation. This study showed that after hESC transplantation, rats injected with the Htt virus showed significant behavioral improvements in behavioral tests. SPION-labeled HESCs migrated with a hypointense signal on MRI. These results suggest that hESC transplantation may treat motor dysfunction in Huntington's disease (Res. 2009).

In turn, Wenceslau et al. also studied the therapeutic potential of human immature dental pulp stem cells (hIDPSC), a particular type of MSC derived from the neural crest, in a mouse model in the treatment of HD. Two doses of hIDPSCs were administered intravenously in a subacute rat model induced by 3-nitro propionic acid (3NP). They also demonstrated the homing of hIDPSCs in the striatum, cortex, and subventricular zone using specific markers for human cells. Thirty days after the administration of hIDPSCs, the cells found in the brain still exhibited the characteristics of undifferentiated MSCs. Quantitative immunohistochemical analysis revealed a significant increase in BDNF, DARPP32, and D2R-positive cells in the striatum and cortex in the groups that received hIDPSCs. The differences were more pronounced in animals that received only one administration of hIDPSC. These results suggest that intravenous administration of hIDPSCs can restore the expression of BDNF, DARPP32, and D2R, promoting neuroprotection and neurogenesis (Islam et al. 2021). Other scientists have also reached similar conclusions, conducting analogous studies on mouse models (Wenceslau et al. 2022).

Dey et al. (2010) studied the effects of bone marrow mesenchymal cell transplantation in YAC128 HD mice. The study used mice genetically modified to overexpress BDNF or nerve growth factor (NGF). It was these mice that, when given the cells, showed significant motor improvements. A more sustained response was demonstrated in mice with BDNF overexpression than in those with NGF. In addition, transplants with BDNF expression reduced neuronal loss (Eskandari et al. 2021).

Using umbilical cord-derived mesenchymal stem cells (MSCs) offers several therapeutic opportunities. This minimizes the risk of immune rejection and simultaneously solves the ethical problems associated with fetal tissue transplants as therapies for neurodegenerative diseases. After MSC transplantation, reduced loss of medium spiny neurons, reduced striatal atrophy, decreased Htt aggregation, and stimulation of endogenous neurogenesis were observed. Despite these promising results, the observed behavioral improvement was not sustained over time (Kim et al. 2021a, b, c), possibly due to the rapid disease progression observed in R6/2 mice.

Al-Gharaibeh et al. used YAC128 mice to study the potential effects of transplantation of induced pluripotent stem cell-derived neural stem cells (iPS-NSCs) (Fink et al. 2013). The iPSC cells were differentiated into neural stem cells (NSCs) in vitro and transplanted into the striatum. Transplantation of iPS-NSCs reduced neuronal loss and increased BDNF and tropomyosin receptor kinase (BTrkB) levels in the striatum. This improved the motility of the mice.

Lee et al. found that exosomes from adipose tissue-derived MSCs (ADMSCs) from R6/2 mice could regulate the HD model's pathogenic features. Immunocytochemistry results showed that ASC-exo (exosomes isolated from adipose stromal/stem cell) significantly reduces mHtt aggregates in mouse neuronal cells. The Western blot result further confirmed the reduction in mHtt aggregates and abnormal apoptotic protein levels in mice treated with ASC-exo (Al-Gharaibeh et al. 2017).

Reidling et al. transplanted hNSCs into the R6/2 and Q140 HD knock-in mice striatum. In R6/2 mice, the grafts were shown to integrate with existing neuronal circuitry, inhibit synaptic changes and improve motility. In Q140 knock-in mice, they improved cognitive impairment in late-stage disease. Moreover, human embryonic neural stem cells increased BDNF expression in both models (Lee et al. 2017).

One clinical trial [NCT032535] has ended, and two [NCT02728115; NCT04219241] are ongoing. They use stem cell therapy in Huntington's disease—Cellavita HD at two doses: a lower dose of 1 × 10^6 cells/weight and a higher amount of 2 × 10^6 cells/weight range per administration. This research aims to prove the therapy's efficacy and safety and develop an appropriate therapeutic dose.

Despite the promising results of animal trials and those clinical trials that have been completed to answer whether stem cell therapy is effective and safe for HD patients, much work still needs to be done. First, it is impossible to translate the results achieved in mouse models to humans directly. In addition, clinical trials are ongoing, and we still have to wait for their final results, especially regarding the therapy's safety. Indeed, one of the problems of clinical trials is the small number of people qualified for trials and the need for uniform trial protocols. In the future, it will be necessary to create a validated research protocol and conduct numerous clinical trials to understand the possibilities of cell therapy in HD fully.

### Alzheimer's Disease

Alzheimer's disease (AD) is a common neurodegenerative disease characterized by the pathological presence of β-amyloid plaques and neurofibrillary tangles.The accumulation of β-amyloid fuels many further components of the disease, including the development of neurofibrillary tangles, loss of neurons and synapses, and cognitive dysfunction. It is the mostcommon form of neurodegenerative dementia, accounting for 50–70% of these cases. The number of people living with dementia worldwide was estimated at 46.8 million in 2015 and is projected to reach 131.5 million by 2050 (Reidling et al. 2018). AD is classified as familial AD (fAD) or sporadic AD (sAD), with fAD manifested mainly by mutations in one of three genes: amyloid -β precursor protein (APP), presenilin 1 (PSEN1), and presenilin 2 (PSEN2). Each of them encodes their respective proteins (Prince et al. 2016; Zhang et al. 2020; Zhan et al. 2017). Aging is also a significant factor in the development of neurodegenerative disorders. This process leads to a progressive loss of neurons, changes in the central nervous system, impaired vascular integrity, and decreased levels of neurotransmitters. Clinically, this results in a lack of attention, reduced ability to learn and remember, and decreased speed of decision-making, motor coordination, and sensory perception. In addition, there is impairment of intellectual abilities and memory loss, which are severe enough to interfere with the patient's and their caregiver's daily life. The mechanisms underlying Alzheimer's disease, however, remain unknown. Current drug treatment approaches only alleviate symptoms, not treat the underlying cause of the disease.Three cholinesterase inhibitors (donepezil, galantamine and rivastigmine) and one N-methyl-D-aspartate receptor antagonist (memantine) are used in the European Union (Filadi and Pizzo 2019). A fifth treatment regimen, consisting of a fixed-dose combination of donepezil and memantine, has also been approved for treating patients with moderate-to-severe, donepezil-stabilized AD dementia (Cummings et al. 2019). Unfortunately, most therapeutic efforts are unsuccessful; AD is one of the least-served therapeutic areas with pharmacotherapy. Research and development of cellular therapies for treating AD have mainly focused on removing Aβ deposits in the brain. Unfortunately, such approaches have thus far failed in late-stage clinical trials. Still, stem cell-based therapies could intervene at multiple subsequent stages of this disease (e.g., synaptic dysfunction, gliosis, inflammation) (Namzaric 2016). A selection of these is shown in Table 5. Currently, the primary sources of stem cells include neural stem cells and mesenchymal stem cells.

**Table 5:** Selected clinical trials for AD

Alzheimer’s disease (AD)				
Year	Animal model	Type of cells	Therapeutic effect	Literature
2011 2012 2021	Human	MSCs	1. Changes in the AD status, 2. Many side effects of the therapy were also noticed	[NCT01297218, NCT01696591, NCT02600130]
2013	Tg2576 mouse model	Adipose derived human stem cells (hASCs)	1. After 12 days after cell administration, strong fluorescence signals were noted in mice’s brains	Hayashi et al (2020)
2014	Tg2576 mouse model	Adipose derived human stem cells (hASCs)	1. Improved memory impairment and neuropathology after intracerebrally therapy	Ha et al. (2014)
2014	Human	Human umbilical cord blood-derived mesenchymal stem cells (hUCB-MSCs)	1. Therapy was feasible, relatively and sufficiently safe, and well tolerated	[NCT02054208]
2017	Human	Autologous hematopoietic stem cell transplantation (AHSCT)	1. Half of the patients with the predominant progressive form survived progression-free for 5 years after transplantation, 2. A patient profile has been created that has a better chance of survival without neurological progression	Chang et al. (2014)
2017	Human	Human iPSCs	1. Increased salivary β (Aβ42) levels	Muraro et al. (2017a)
2019	Mouse AD model (APPswe / PS1dE9)	BM-MSC-EVs	1. Reduce the level of Aβ and dystrophic neurites in the cortex and hippocampus, 2. Degradation of Aβ plaques and reduction the amount of Aβ deposits	Muraro et al. (2017b)
2020	APP / PS1 AD mouse model	NSC-EVs	1. Increased activation of SIRT1, levels of memory-related synaptic proteins and morphology in the cerebral cortex, 2. Reduced oxidative damage and the level of pro-inflammatory cytokines and microglia markers in the cerebral cortex	Chen et al. (2001)
2018	Human	Autologous bone marrow-derived stem cells (BMSC)	1. No results yet	[NCT03724136]
2021	β-amyloid-induced AD rat model	human umbilical cord mesenchymal stem cells (hUMSCs) and mesenchymal adipose tissue stem cells (hAD-MSCs)	1. Transplantation of hUMSCs and hAD-SCs significantly reduced the apoptosis rate of hippocampal neurons 2. Transplantation of MSCs resulted in increased expression levels of synaptic (synaptophysin) and neurogenic markers 3. Intravenous injection of both SCs ameliorated learning and cognitive impairment induced by β-A42 injection	Doshmanziari et al. (2021)

#### NSCs in AD

As a novel therapeutic strategy, neural stem cell (NSC) transplantation targets neuronal networks and pathological proteins, resulting in positive behavioral and microenvironmental outcomes. In short, most conventional drug therapies act only on the microenvironment. As pluripotent stem cells, NSCs can self-renew and differentiate into different cell types, such as neurons and glial cells (Chen and Blurton-Jones 2012; Massirer et al. 2011). NSCs can be derived from brain tissue, can be genetically reprogrammed from somatic cells (Martinez-Morales et al. 2013; Shahbazi et al. 2018), and can even be differentiated from embryonic stem cells (ESCs) and iPSCs (Hermann and Storch 2013). In adults, NSCs are located in the subventricular zone (SVZ) and hippocampus (Wen and Jin 2014; Guo et al. 2012; Lee et al. 2008). Interestingly, neuralstem cells (NSCs) can express high levels of neurotrophic factors, including BDNF and NGF, which regulate synaptic plasticity and provide potent neuroprotective and neurotrophic activities.

Different stem cell populations can promote anti-inflammatory signaling that slows disease progression. Genetic modification of stem cells can also be used to simultaneously target Aβ and tangle pathology or enhance neurotrophic and neuroprotective capabilities. NSCs can be used to provide a practical approach to delivering therapeutic Aβ-targeted proteins to the brain. Complementing transplantation with therapies such as passive Aβ immunization would be appealing. Thus, NSC-mediated delivery of neurotrophins may indeed play an important role in regulating cognition and synaptic plasticity in AD models, although additional mechanistic studies are required (Hayashi et al. 2020). Zhang et al. (2017) also demonstrated that NSC transplantation can rescue cognitive and synaptic deficits in specific regions. Recovery of neuronal function is crucial for learning and memory in AD mouse models (Chen and Blurton-Jones 2012).

As a novel therapeutic strategy, neural stem cell (NSC) transplantation targets neuronal networks and pathological proteins, resulting in positive behavioral and microenvironmental outcomes. In short, most conventional drug therapies act only on the microenvironment. As pluripotent stem cells, NSCs can self-renew and differentiate into different cell types, such as neurons and glial cells (Chen and Blurton-Jones 2012; Massirer et al. 2011). NSCs can be derived from brain tissue, can be genetically reprogrammed from somatic cells (Martinez-Morales et al. 2013; Shahbazi et al. 2018), and can even be differentiated from embryonic stem cells (ESCs) and iPSCs (Massirer et al. 2011; Hermann and Storch 2013). In adults, NSCs are located in the subventricular zone (SVZ) and hippocampus (Wen and Jin 2014; Guo et al. 2012; Lee et al. 2008). Interestingly, neural stem cells (NSCs) can express high levels of neurotrophic factors, including BDNF and NGF, which regulate synaptic plasticity and provide potent neuroprotective and neurotrophic activities.

**Fig. 4:**
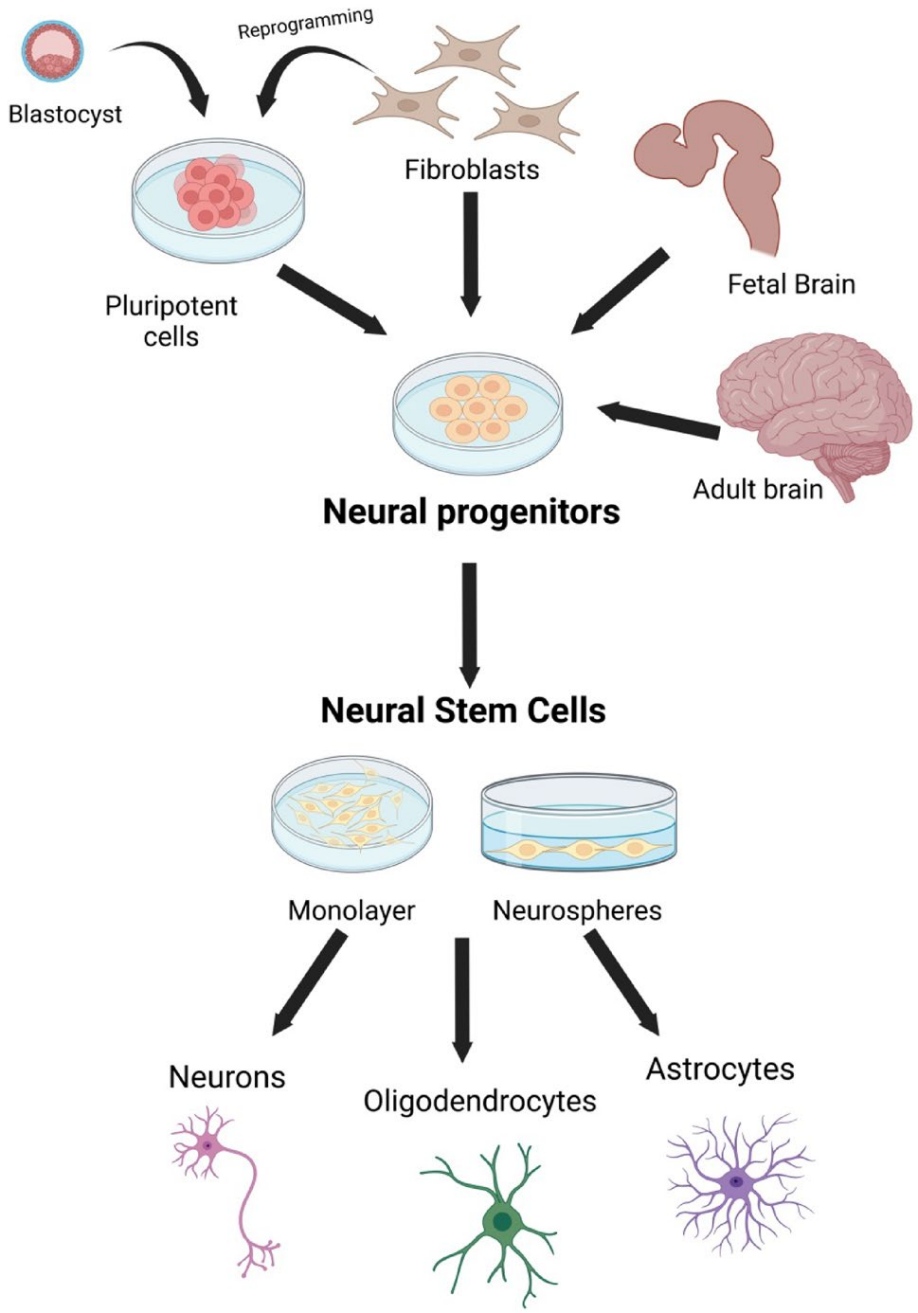
Sources and in vitro expansion protocols for generation and expansion of neural stem cells. Neural stem cells can be isolated from different regions of the brain of rodents (mice and rats) and humans at several developmental stages, as well as from budding regions of the adult brain. A common approach is to grow NPCs into neurospheres. These are free-floating aggregates of neural progenitors, each theoretically originating from a single NPC. Their generation relies on microdissection of neural tissue followed by exposure to defined mitogen-supplemented media. In one such method, primary cells are seeded in low-attachment flasks containing serum-free medium supplemented with EGF and/or FGF-2. Under these conditions, differentiated or differentiated cells are said to die and NSCs respond to mitotic agents, divide and form buoyant aggregates (primary neurospheres) that can dissociate and replate to produce secondary neurospheres. This process can be repeated multiple times in succession to expand the NPC population. Complementing neurosphere culture is adherent culture, in which cells can be more easily monitored and have better access to growth factors. Under these conditions, cells divide symmetrically and retain their tripotential differentiation capacity. Adherent culture protocols have been shown to allow cultures with fewer differentiated cells compared to neurosphere assays, which assume cell–cell contacts and uneven mitogen exposure to stimulate a differentiation program

In 2015, Ager et al. human central nervous system stem cells (hCNS-SCs) were first introduced into 3xTg mice. Transplanted hCNS-SCs differentiated into NSCs, immature neurons, and glial cells with increased synapse density. The Morris-Water maze and novel object recognition tests showed improved memory consolidation, although low levels of Aβ and tau protein remained unchanged. Although encouraging, these results suggest that specific differentiation to neuronal cell lines alone contributes little to cognitive recovery and that hCNS-SC transplantation can only be used to reverse symptoms (Zhang et al. 2017). The beneficial effects of NSCs are due less to the modulation of pathological protein levels and more to increased synaptic density, restoration of local neuronal numbers, and/or increased neurotrophic factors. The question is how long this can last while the levels of the pathological protein remain the same. It is also interesting to understand the possible role of NSCs in aggregating damaged proteins through mediation by glial cells, inflammation, and synaptic rescue. Although specific challenges remain, NSCs may be essential in advancing AD treatment (See Fig. 4).

#### MSCs in AD

MSCs can transform into various cell types in bone marrow, adipose tissue, lung, liver, and umbilical cord (Phinney and Prockop 2007). Isolated MSCs can proliferate and differentiate into osteoblasts, adipocytes, and pancreatic islets (Dominici et al. 2006). Chronic inflammation plays a vital role in AD.

MSCs can transform into various bone marrow, adipose tissue, lung, liver, and umbilical cord cell types. Isolated MSCs can proliferate and differentiate into osteoblasts, adipocytes, and pancreatic islets. Chronic inflammation plays a vital role in AD.

In particular, mesenchymal stem cells (MSCs) have been shown to induce the expression of anti-inflammatory factors such as interleukin-10 and prostaglandin. However, it is important to determine the mechanism and whether MSC transplantation alters inflammation directly or as a result of tissue damage (Ager et al. 2015).

In vitro, human MSCs can dramatically increase the number of neurons in the hippocampus and induce NPCs to become neurons via the Wnt signaling pathway. Additionally, human MSCs can decrease the amount of Aβ42 by promoting autophagy in vitro and in vivo. Further, transplantation of Bone Marrow Mesenchymal Stromal Cells (BMMSCs) or human umbilical cord blood-derived MSCs into AD mouse models' lateral ventricles or hippocampi improves memory and spatial learning by decreasing Aβ42 levels and increasing neuronal survival. Similarly, autologous BMMSCs have been successfully transplanted into the brains of patients with ischemic disease. It reduced the infarct size and improved functionality.The possible role of human umbilical cord mesenchymal stem cells (hUMSCs) and mesenchymal adipose tissue stem cells (hAD-MSCs) in neurogenesis and synaptic function was investigated using a β-amyloid-induced AD rat model. Transplantation of MSCs reduces β-amyloid deposition in the hippocampus of AD rats. Transplantation of hUMSCs or hAD-SCs significantly reduced the apoptosis rate of hippocampal neurons.

Furthermore, transplantation of MSCs resulted in increased synaptic (synaptophysin) and neurogenic markers expression levels. Therefore, intravenous injection of hUMSCs and hAD-MSCs is a safe approach to improve synaptic function and neurogenesis by increasing synaptic protein expression in Alzheimer's disease models. Intravenous injection of both SCs ameliorated learning and cognitive impairment induced by β-A42 injection (Chen and Blurton-Jones 2012).

The utilization of MSCs has been touted as a promising approach for treating stem cell disorders compared to that of NSCs. However, many types of MSCs have harmful side effects in use. The extraction and cultivation of BMMSCs are complicated, which limits their utilization in clinical investigations. Despite these shortcomings, in recent years, ADSCs and umbilical cord blood-derived MSCs have been demonstrated to be novel options for treating AD (Doshmanziari et al. 2021).

#### iPSCs in AD

The advent in the last decade of techniques for generating human induced pluripotent stem cells (iPSCs) and differentiating them into various cell types of the body, including brain cells, has ushered in a new era of neurodegenerative disease research. Using iPSCs as an alternative avoids many ethical concerns associated with embryonic stem cells. Nonetheless, to what extent these iPSCs are uniformed in quality remains in question. In addition, iPSC-based systems and genome editing tools are crucial to understanding the role of many new genes and mutations that modify Alzheimer's disease risk.

The CRISPR/Cas9 system is revolutionary, enabling targeted mutagenesis and base pair resolution editing of eukaryotic genomes. Although care must be taken to exclude target mutations generated by CRISPR/Cas9 mutagenesis, this technique allows the effects of target mutations to be studied in an identical (isogenic) genetic background. Genome editing can introduce disease-related mutations into iPSC lines from healthy individuals or correct mutations in cell lines from diseased patients. Astrocytes provide physical, energetic, metabolic, and trophic support to neurons and other brain cells, the most abundant cell type in the human brain. In addition, astrocytes are a significant source of cholesterol and other lipids critical for many cellular functions, as well as lipoproteins such as APOE, which are thought to be essential regulators of Aβ clearance and degradation in the brain. Many different protocols have been developed to differentiate iPSCs into astrocytes. Astrocytes derived from iPSCs have been shown to have increased release and decreased uptake of Aβ42, altered Ca2 + homeostasis, increased production of reactive oxygen species, modified out of cytokines, and impaired fatty acid oxidation.

**Fig. 5:**
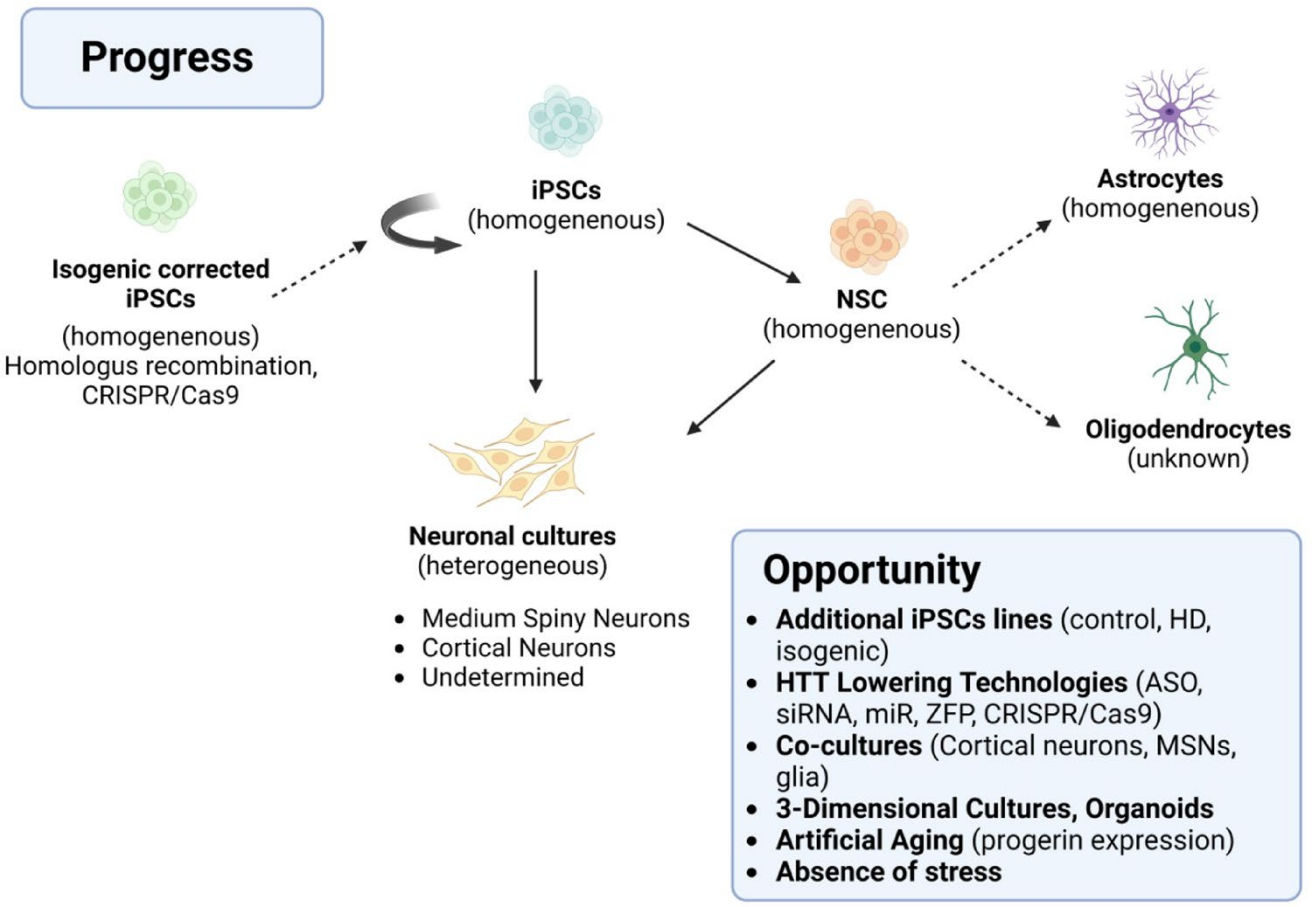
Induced pluripotent stem cells (iPSC) in Huntington’s disease research. This figure shows the neural lineage cell types that can be distinguished from iPSCs. The relative purity achievable by different cultures is indicated in parentheses (homogeneous or heterogeneous). Differentiated cultures are rarely 100% pure, but can reach 95% homogeneity depending on the cell type. Neuronal cultures often contain large numbers of glial cells, including nestin-positive neural stem cells (NSCs), astrocytes, and oligodendrocytes (heterogeneity). Dashed arrows indicate areas requiring further research

Further improving iPSC differentiation protocols, expanding the repertoire of brain cell subtypes that can be generated, and developing more complex 3D coculture systems for modeling brain development and disease remain essential goals for iPSC researchers. These efforts aim to improve the ease and speed of differentiation protocols and the differentiated cells' quality, purity, and maturity (Mazini et al. 2019) (see Fig. 5).iPSCs in AD models have successfully mimicked the pathological state, which can be used to investigate new treatments, such as combining bromocriptine, cromolyn, and topiramate as an anti-Aβ cocktail (Penney et al. 2020) and secretase inhibitors (Kondo et al. 2017; Yagi et al. 2011). These medications prevent the production of Aβ, and as a result, the toxic level of Aβ is decreased. Specifically, treatment with the anti-Aβ cocktail reduces toxic Aβ levels by over 60%. This same effect is achieved by treatment with inhibitors (Penney et al. 2020). Additionally, although the mutations in FAD and sporadic AD neurons differ, a decrease in Aβ levels was observed in both cases (Kondo et al. 2017; Yagi et al. 2011). The administration of antibodies to Aβ to neurons derived from iPSCs identified Aβ as a cause of increased tau phosphorylation. This outcome further supports established principles and provides insights into drug development (Yagi et al. 2011; Muratore et al. 2014).

#### Other Stem Cells in AD

Scientists administered intravenous adipose-derived human stem cells (hASCs) labeled with fluorescent magnetic nanoparticles. After 12 days after cell administration, strong fluorescence signals were noted in Tg2576 brains ([Hayashi et al. 2020). One year later, using the same model, hASC was also administered intracerebrally, significantly improving memory impairment and neuropathology (Ha et al. 2014).

The resultsafter autologous hematopoietic stem cell transplantation (AHSCT) show that nearly half of the patients with the predominant progressive form survived progression-free for 5 years after transplantation. In addition, a patient profile has been created that has a better chance of survival without neurological progression. Age is essential—young, the form of the disease—recurrent, current treatment—no more than 2 disease-modifying treatments and the lack of a high level of disability (Chang et al. 2014). In another study by the team mentioned above, scientists examined human iPSCs produced from skin cells and fibroblasts that can differentiate into neuronal cells and increase salivary β (Aβ42) levels (Muraro et al. 2017a).

Extracellular vesicles (EVs) derived from bone marrow-mesenchymal stem cells (BM-MSC-EVs) were injected into the cortex of a new mouse AD model (APPswe/PS1dE9). They turned out to reduce the level of Aβ and the number of dystrophic neurites in the cortex and hippocampus. MSC EVs also contain the enzymes neprilysin, a protease, which degrades Aβ plaques and consequently reduces the amount of Aβ deposits (Muraro et al. 2017b).

Another study using EVs derived from NSC was carried out on the APP/PS1 AD mouse model. After 5 weeks of therapy, increased activation of SIRT1 in the cerebral cortex was demonstrated. Its activation increases memory and the formation of dendritic spines (Elia et al. 2019). Increased memory-related synaptic proteins and improvements in synaptic morphology in the cerebral cortex have been observed. On the other hand, oxidative damage and the level of pro—inflammatory cytokines and microglia markers in the cerebral cortex were reduced co mpared to the control group (Chen et al. 2001).

**Fig. 6:**
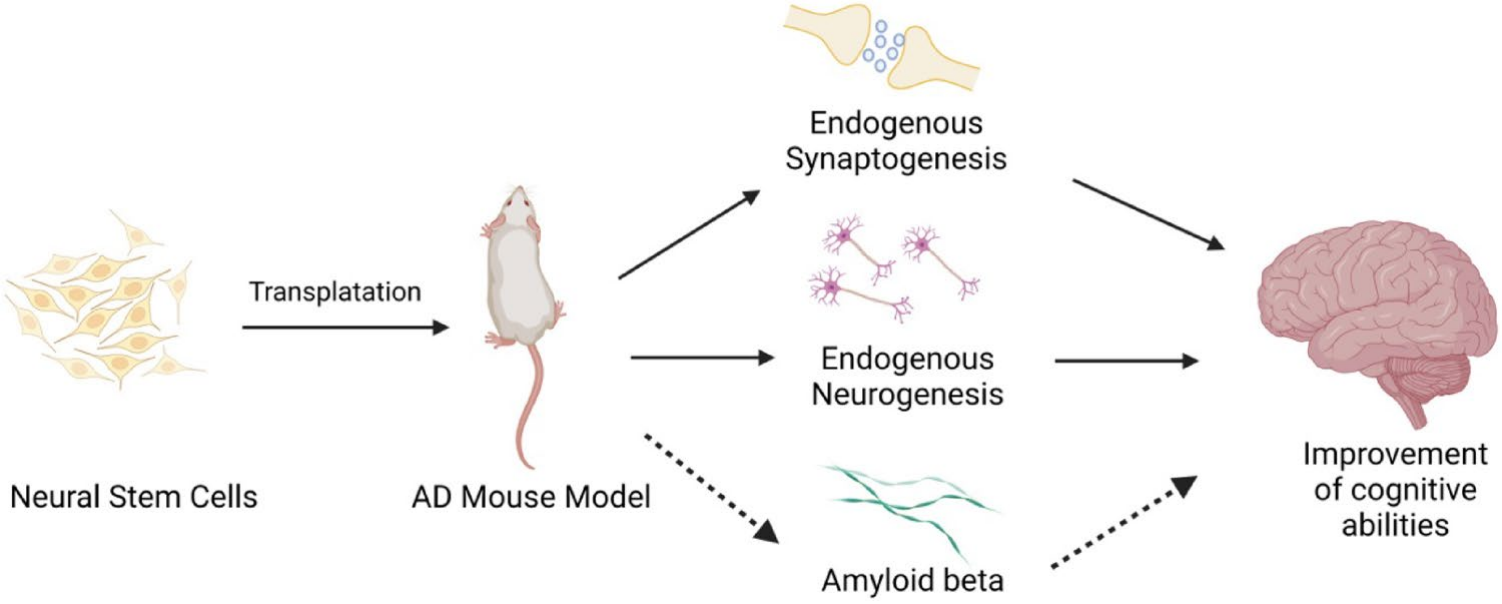
Pathways for neural stem cell transplantation and mechanisms for restoring cognitive dysfunction. Transplantation of neural stem cells triggers endogenous synaptogenesis and endogenous neurogenesis, affecting recovery of cognitive impairment. The limited causal relationship between amyloid-beta (dashed arrows) and neural stem cells rules out any link between behavior and amyloid-beta aggregation

Transgenic and knockout mouse models of APP, presenilin, and ApoE have taught us much about the function of these genes and their role in AD pathogenesis. Accordingly, recently were generated hESC clones that overexpress wild-type or mutant forms of human APP. Surprisingly, it was discovered that all of the resulting APP hES clones rapidly (in as many as 5 days, whereas with standard methods, to obtain the same amount of neuronal differentiation, one has to wait about 3 weeks) and spontaneously differentiate toward the neuronal lineale (Li et al. 2020) (see Fig. 6).

#### Clinical Trials in AD

The clinical trial [NCT02054208] involved nine patients with mild to moderate dementia in Alzheimer's disease. Four weeks before MSC administration, the Ommaya reservoir was implanted into the patient's right lateral ventricle. Three patients received low-dose, and six received high-dose human umbilical cord blood-derived mesenchymal stem (hUCB-MSCs). All nine patients received three repeat injections of MSCs (at 4-weeks intervals). These patients were followed up to 36 months. The results showed that the triple injection of hUCB-MSCs into the lateral ventricle via the Ommaya reservoir was feasible, relatively and sufficiently safe, and well tolerated (Chen and Blurton-Jones 2012). A clinical trial [NCT03724136] is currently underway using autologous bone marrow-derived stem cells (BMSC). The use of near-infrared in conjunction with the BMSC will also be assessed. The therapy is intended to improve the cognitive functions that occur not only in AD but also in other dementias.

Stem cell therapy may be combined with other treatments, such as gene therapy or drug therapy, to enhance its effectiveness. Ongoing clinical trials are expected to yield more information about the safety and efficacy of stem cell therapy for Alzheimer's disease, paving the way for the broader adoption of this innovative approach. It's important to note that stem cell therapy for Alzheimer's disease is still in the early stages of research. It may be several years before it becomes a widely available treatment option. However, with continued research and development, stem cell therapy has the potential to significantly improve the lives of those living with Alzheimer's disease in the future.

## Conclusions

Stem cell research offers great hope for improving people's health with neurodegenerative diseases. Despite many studies in animal models and clinical trials, cell therapy must be refined to increase its effectiveness in the human body. Despite the need to continue research, the results achieved so far by scientists carry great hopes for using stem cell therapy to treat neurodegenerative diseases. The most important conclusions that arise after analyzing the literature:

1. MSC cells slow down ALS progression and show early promising signs of efficacy. MS therapy with hematopoietic stem cells (HSC) inducted significant recalibration of pro-inflammatory and immunoregulatory components of the immune system. Bone marrow-derived MSCs induced to secrete neurotrophic factor (NTF), increased CSF neurotrophic factors. The results show that all cell transplantations are safe and show early promising signs of efficacy.
2. In MS, therapy with MSC cells has shown to be more potent than therapy with immunomodulatory drugs and to prevent excessive glial scarring in the CNS. In most patients increase in the percentage of regulatory T cells, decreased proliferative responses of lymphocytes, and the expression of CD40 + , CD83 + , CD86 + , and HLA-DR on myeloid dendritic cells, thanks to which inflammation is suppressed. Hematopoietic stem cell (HSC) therapy significantly reduces the number and volume of gadolinium-enhancing lesions on MRI, resulting in prolonged disease progression.
3. iPSC cells allow for accurate PD modeling. They are patient-specific and therefore minimize the risk of immune rejection and, in long-term observation, did not form any tumors in the brain. An animal model demonstrated increased dopaminergic neurons midbrain. Moreover, histological studies have shown that mature dopaminergic neurons extend dense neurites into the host's striatum. This results in improved movement.
4. MSCs have a significant role in the treatment of HD. Cells have an impact on motor improvements and stimulation of endogenous neurogenesis. Also, MSCs reduce neuronal loss, loss of medium spiny neurons, striatal atrophy, and huntingtin aggregation. Moreover, adipose tissue-derived MSCs also reduce levels of abnormal apoptotic protein.
5. BM-MSC-EVs cells are widely used to treat AD by reducing the level of Aβ and dystrophic neurites in the cortex and hippocampus, degradation of Aβ plaques, and reducing the number of Aβ deposits. These factors all lead to an increase in improved memory and learning abilities. Adipose-derived human stem cells (hASCs) also decrease neuropathology after intracerebral therapy.
6. Stem cells—NSC is increasingly used in HD, ALS, and AD. NSCs can self-renew and differentiate into different cell types, such as neurons and glial cells. However, it is necessary to obtain uniform protocols for obtaining these cells and research confirming the safety of their use.

On the other hand, there are several difficulties associated with using stem cells for these conditions:

1. Limited availability of stem cells: The number of stem cells that can be collected from a person is limited, and the collection process can be invasive and risky.
2. Difficulty reaching affected areas: Delivering stem cells to the appropriate brain regions is challenging because it requires crossing the blood–brain barrier and navigating through complex neural networks.
3. Safety issues: There are potential risks in stem cell transplantation, including the possibility of immune rejection and the risk of tumor formation.
4. Lack of understanding of disease pathology: The underlying causes of neurodegenerative diseases are complex and not fully understood, making it difficult to predict how stem cells in affected brain tissue will behave.
5. Ethical considerations: The use of stem cells raises ethical questions about the origin of cells, especially in the case of embryonic stem cells.

Despite these challenges, researchers continue to investigate the potential of stem cells as a treatment for neurodegenerative diseases, and ongoing clinical trials are exploring the safety and efficacy of various approaches.

## Data Availability

Not applicable.
